# “Happy Farmers” in Volta Delta, Ghana? Exploring the Relationship between Environmental Conditions and Happiness

**DOI:** 10.1007/s11205-025-03632-8

**Published:** 2025-07-14

**Authors:** Laurence Cannings, Craig W. Hutton, Alessandro Sorichetta, Kristine Nilsen

**Affiliations:** 1 School of Geography and Environmental Science, University of Southampton, Southampton, SO17 1BJ UK; 2 Dipartimento Di Scienze Della Terra “A. Desio”, Università Degli Studi Di Milano, Via Mangiagalli 34, 20133 Milan, Italy; 3 Department of Social Statistics and Demography and WorldPop, School of Geography and Environmental Science, University of Southampton, Southampton, SO17 1BJ UK

**Keywords:** Happiness, Environment, Wellbeing, Hazard, Landscape, Agriculture

## Abstract

Communities’ wellbeing in rural lower-middle-income countries is interlinked with climate and landscape characteristics. Rural inhabitants are often assumed to be “happy farmers”, content with their livelihoods and social connections, despite the financial and material insecurities associated with their fragile environments. However, is this assumption an accurate reflection of reality? This study explores relationships between environmental conditions and subjective wellbeing in Volta Delta, Ghana. Subjective wellbeing is captured through a life domains happiness measure, calculated using the “Deltas, Vulnerability and Climate Change: Migration & Adaptation” survey dataset. A binary logistic model evaluates associations between low happiness, and environmental and control characteristics constructed from survey and remote sensing datasets. The quantitative approach supports the “happy farmer” identity, with lower probabilities of low happiness amongst rural households with a strong attachment to agricultural landscapes. However, the limited availability of permanent employment could offset these subjective benefits. Nevertheless, happiness is not a substitute for objective wellbeing, often defined through monetary wealth; therefore, sustainability policy should not be discouraged from providing tangible support to vulnerable communities. Volta Delta consists of varying landscapes, with model results also illustrating lower happiness within coastal locations, potentially linked to fears of hazards, restricted natural resource governance, and threats to intergenerational land and livelihoods. This study highlights the key role of environmental conditions in potentially influencing subjective wellbeing. Exploring relationships with subjective outcomes ensures sustainability policy captures non-tangible outcomes and feedback effects, which, if incorporated alongside objective targets, can ensure all costs, benefits and challenges are accounted for.

**Supplementary Information:**

The online version contains supplementary material available at 10.1007/s11205-025-03632-8.

## Introduction

Wellbeing and environment are inherently connected; therefore, understanding the complex relationships between human needs and supporting ecosystems is crucial in fostering sustainable development. However, due to the poor integration of wellbeing research within the field of sustainability (Helne & Hirvilammi, 2015; Voukelatou et al., 2021), there is limited empirical evidence supporting environment-wellbeing relationships (Schleicher et al., 2018). In particular, fewer studies explore environmental associations with subjective wellbeing (SWB) (Brown et al., 2021; Lawrance et al., 2021). SWB captures individuals’ internal assessments and cognitive judgments, generated from “affects”, including emotions and moods, and “thoughts” regarding individuals’ life evaluations in relation to socially-constructed expectations and aspirations (Veenhoven, 2009). There is no consensus on how SWB should be defined or measured, with interpretations situated across three categories (Table 1).

**Table 1:** Different conceptual theories of subjective wellbeing, adapted from Dolan et al. (2011)

Theory	Description
Evaluation	• Most common approach in social research and policy • Focused on general assessments of “life overall” or different life domains such as health, job security and education • Often specified by whether individuals’ desires are fulfilled (Ravallion, 2014)
Experience	• Measures the balance of positive and negative emotions • A hedonistic approach, meaning wellbeing is the experience of more pleasure than pain (Schleicher et al., 2018)
Eudaimonic	• Measures inherent psychological positives that all individuals strive towards, the needs that give life meaning (Addai et al., 2014) • Non-substitutable elements which are important to individuals’ identities and livelihoods (Schaafsma, 2019) • Wellbeing may not necessarily result from pleasure; instead, wellbeing is achieved by acting virtuously and growing as a human (Ayerakwa et al., 2015) • Deci and Ryan (2012) record three universal elements: autonomy, relatedness (social connection), and competence (mastery of skills/environment)

Sulemana et al. (2016) suggest that studies on environment-SWB associations predominantly focus on the psychological impacts of global environmental challenges in high-income countries (HICs), whereas in lower-middle-income countries (LMICs) SWB is more influenced by local, rather than global, environmental challenges. The limited focus on SWB in LMICs could be attributable to Maslow’s “hierarchy of needs”, where communities prioritise immediate material needs, rather than “bonus” psychological needs encompassed within SWB (Chumo et al., 2023). Furthermore, many studies focus on singular, objective outcomes, such as monetary poverty, and implicitly assume they capture the totality of wellbeing (Barbier & Hochard, 2018). Despite certain studies suggesting the fulfilment of objective needs constitutes the foundation of SWB in LMICs (Guillen-Royo et al., 2013; Nanor et al., 2021), these approaches potentially omit important elements of human wellbeing (Copestake, 2008) such as social relationships, cultural identity and purpose, which are unattainable through market channels (Ward & King, 2016).

The importance of encompassing happiness (SWB) within sustainable development is underscored by research showing that the psychological impacts of climatic hazards often outweigh the physical effects (Hayes et al., 2018). Therefore, understanding SWB determinants, and acknowledging the need for SWB within development policy, can increase the scope of potential wellbeing improvements. Ignoring SWB can also result in the associated hidden costs being overlooked; for example, low happiness can negatively impact employment and productivity (Knapp, 2003; Trautmann et al., 2016). These tangible implications may further lower SWB in contexts such as Ghana, where a “good life” is growth-orientated and related to material wealth (Dzokoto et al., 2019). Additionally, with increasing economic, social and environmental challenges, communities must possess a “resiliency culture” (Addai et al., 2014; p.874). Happy communities are resilient communities; therefore, by investigating the driving factors behind actors’ happiness, and understanding the mechanisms which keep individuals unified and at peace, policies can be supported to generate and maintain greater environmental resiliency and sustainable development (O'Brien, 2013).

Multiple SWB and happiness measures are frequently reported; however, there is great variability in the samples, methodologies and results. Taking Ghana as an example, the World Happiness Report 2024 placed Ghana 120/143 globally, based on responses to the Cantril ladder question^1^ (Helliwell et al., 2025), whereas the Gallup (2014) “State of Global Well-being report” ranked Ghana 136/155; with particularly low scores relating to purpose, physical and financial wellbeing. In contrast, the World Database of Happiness 2003 ranked Ghana 7 th for life satisfaction (Veenhoven, 2005), and the World Value Survey 2010, which captures individuals' values and social changes, positioned Ghana 51/97 (Dzokoto, 2012). These varying results justify the need to investigate SWB within a specific relational context (White, 2016), with this study focusing on a vulnerable delta in Ghana. Despite focusing on a specific case study site, the methods and concepts used can be replicated in other locations to identify similarities or context-specific differences, and support policy in addressing the wide-ranging challenges surrounding climate hazards and natural resource access.

This paper explores modelled relationships between environmental conditions and low happiness in Volta Delta. “Environmental conditions” include climate hazards, such as droughts and floods, and landscape characteristics such as landcover classifications, which implicitly contain information on livelihoods, ecosystem services and social norms (Forkuo et al., 2021). A binary logistic regression model, incorporating survey and remote sensing datasets, is constructed to quantify local environment-wellbeing relationships. A broader suite of wellbeing determinants, such as employment status, are incorporated as “control” characteristics, and are interpreted to function within the broader context of environmental vulnerability.

The focus on environmental conditions within deltas is due to them being “one of the most vulnerable environments threatened by climate and human-induced changes” (Foufoula-Georgiou et al., 2013; p.4). Due to the combination of high population densities, economic potential, environmental vulnerability, and high dependency upon ecosystem services for human and material wellbeing (Kuenzer & Renaud, 2012), deltas, many of which are located in LMICs, are hotspots for international sustainable development (Foufoula-Georgiou et al., 2013). Furthermore, the myriad of environmental and socioeconomic challenges faced within deltas creates a space for research to explore the multiple mechanisms that may influence different elements of multidimensional SWB. This study contributes to integrating wellbeing research within sustainable development. The novel approach views SWB from a landscape perspective, acknowledging how outcomes can differ spatially across the diverse contexts of a deltaic environment with varying climates, land-use and livelihoods.

Overall, this study aims to address two research questions: (i) what are the recorded levels of happiness in Volta Delta? (ii) how do climate hazards and landscape characteristics associate with recorded happiness outcomes? Investigating these research questions will add to the discussion around the “happy farmer” assumption, where rural inhabitants are depicted as content with their lives despite financial and material challenges (Markussen et al., 2018). Additionally, addressing these questions can highlight the capacity of SWB outcomes to capture broad information on communities’ responses to environmental pressures, and the benefit of exploring less tangible wellbeing conceptualisations when implementing sustainable development policy (Sulemana et al., 2016).

## Background

This section reviews existing literature to outline the benefits and challenges of incorporating SWB within sustainable development policy, followed by an exploration of documented relationships between environmental conditions and SWB.

### SWB in Policy

Quantifying “happiness” from an “evaluation” perspective is the most common approach in social science research and policy (Dolan et al., 2011). However, quantifying complex social phenomena brings challenges; for example, static statistical outputs juxtapose the fluidity of individual and societal processes (Shaffer, 2013). Nevertheless, the importance of simplicity and pragmatism from a policy perspective is recognised. An absence of a consensus on measuring “happiness” should not prevent action. Recent methodological improvements have increased the access to actionable “happiness” metrics amongst policymakers, exemplified in UAE which founded the Global Council for Happiness and Wellbeing “to identify best happiness practices” for government institutions and businesses (Sachs, 2019; p.6).

Incorporating SWB within sustainability research and development policy has multiple benefits. SWB measures create a channel for public voices to be reflected in policy (Diener & Suh, 1997; Dolan & White, 2007; Vaznonienė, 2014), which can improve community buy-in and policymaker accountability (De Schutter, 2021); particularly powerful in Ghana where historically top-down initiatives have neglected local knowledge (Domfeh and Nyigmah Bawole, 2009). Furthermore, Flik and van Praag (1991) state “individuals themselves are the best judges of their own situation” (p.313); therefore, SWB measures can minimise the influence of external assumptions and provide more accurate, context-specific results (Nunan, 2015).

Individuals’ emotions and perceptions, incorporated within SWB, govern behaviour (Alam & Mallick, 2022). Therefore, exploring SWB can enhance decision-makers’ understanding of communities’ priorities and actions, particularly if they do not align with targeted objective outcomes, thereby improving buy-in and policy success. For example, Coulthard (2011) found local investment in fishing livelihoods amongst coastal Indian communities to be prioritised despite fishing livelihoods consistently failing to provide food and financial security, due to the increased dignity, status and SWB received from fulfilling their desired identities.

Designing policy through a “happiness” lens can ensure comparability and collaboration across different actors and departments (Helliwell, 2019), as despite discrepancies over its theoretical underpinnings, as a concept, “happiness” is universally relatable and recognised as innately positive (Dasgupta, 2022). Therefore, despite SWB being a multidimensional, personal outcome, numerical metrics can facilitate its inclusion within spaces historically dominated by quantitative methods, such as sustainability and environmental justice studies (Althor & Witt, 2020), and ensure research findings are practical.

SWB can also be impacted by, and influence the success of, environmental policies. SWB often reduces in the short-term following the implementation of environmental policy due to lower consumption being enforced (Kingdon & Knight, 2006), exemplified by the low global correlation between SWB and SDG12-13 progression^2^ (De Neve & Sachs, 2020). However, van den Bergh (2010) suggests that slowing economic growth due to climate policy may result in negligible reductions in happiness within certain contexts, with the avoidance of extreme hazards, the consequent health improvements, and the capacity for collective environmental action to empower communities (Brown and Kasser, 2005; Lawrance et al., 2021), conversely stimulating long-term SWB improvements (FitzRoy et al., 2012; Qasim & Grimes, 2021). Incorporating SWB initiatives into environmental policies can help mitigate the potential initial decline in wellbeing and create positive feedback effects, with studies illustrating happier people to be more likely to support environmental policies (Gowdy, 2004; Sulemana et al., 2016).

SWB measures beneficially capture wide-ranging information, including emotions towards health, future opportunities and housing (Mahmood et al., 2019; Marks, 2007; Narayan & Petesch, 2002; Ravallion & Lokshin, 2002; Reyes-García et al., 2016; Wang et al., 2020). However, from a policy perspective, the complex information incorporated in SWB can restrict policymakers from pinpointing specific causes and solutions to communities’ challenges (Brüggen et al., 2017).

Comparisons to others, and past periods of time, are key SWB determinants, especially within uncertain environments where changing conditions repeatedly alter natural resource access and financial wealth. Therefore, if policy only monitors success through SWB measures, the “grumbling rich man” could be prioritised over the “contented peasant” (Sen, 1983; p.160), as “happiness is not the result of being rich, but a temporary consequence of…becoming richer” (Inglehart, 1990; p.212). However, happiness should not be considered a substitute for tangible, material support (Kay & Jost, 2003). Defining objectively poor households as “content” or “happy farmers” can romanticise financial poverty from a Western perspective (Crossley, 2012), rationalise inequality and discourage material investments (Davis, 2014). If SWB-based policies fail to recognise how “happy but poor” individuals do not “love squalor, [rather] they make do with what they have” (Dowling & Yap, 2012; p.4), interventions could be de-politicised and aim to change how people view the world, rather than how the world is (White, 2010). Therefore, despite this paper specifically focusing on SWB, further research exploring other wellbeing measures, and their relationship to SWB, is encouraged to ensure all costs, benefits and challenges are accounted for (Clare et al., 2017; Vaznonienė, 2014).

### SWB and Environment

Environmental threats to deltas are primarily driven by anthropogenic temperature increases, which contribute towards prolonged droughts, intensified rainfall, unpredictable monsoon seasonality and rising sea levels (Niang et al., 2014; Raj et al., 2019). However, despite LMICs facing the gravest consequences of global warming due to their greater exposure and sensitivity and lower adaptive capacity (Brown et al., 2007), they are minor contributors. Althor et al. (2016) showed thirty, predominantly tropical, countries to be within the bottom two quintiles for carbon emissions in 2010, yet the top two for climate vulnerability.^3^ Ghana is estimated to experience increased “acute” (highest quintile) vulnerability by 2030. This global disparity in vulnerability is driven by skewed development processes, including uneven (post)colonial investment patterns (Awanyo & Attua, 2018; Kambala, 2022) and natural resource mismanagement (Cardona et al., 2012).

Multiple documented SWB controls within LMICs can be interpreted through an environmental lens. Firstly, Jax et al. (2018) suggest that “caring for nature” is an innate component of eudaimonic wellbeing; therefore, an inability to prevent climate-induced degradation may directly reduce communities’ happiness. Secondly, health is interpreted as the core determinant of SWB in LMICs (Addai et al., 2014; Dzokoto, 2012; Van Praag et al., 2003). Climate hazards are associated with various physical and mental health issues; for example, increases in cholera following flooding in areas with inadequate latrine facilities (Mireku-Gyimah et al., 2018; Osumanu et al., 2019), and issues of anxiety linked to uncertain seasonality and food insecurity (Stokols et al., 2009). Large, abrupt climatic changes can lower SWB by reducing actors’ perceived agency to adapt to unforeseen conditions (Mkrtchyan et al., 2018), whilst also contributing towards a sense of loss and disrupting the “calmness” needed for a “good life” (Osei-Tutu et al., 2020); “when certainties move you feel a loss of control of your life, which is demoralizing” (Jennings & Magrath, 2009; p.12)*.*

Studies also illustrate an association between psychological damage, such as post-traumatic stress,^4^ and being directly and/or indirectly impacted by climate hazards (Doherty & Clayton, 2011; Neria et al., 2008). For example, Sekulova and Bergh (2016) found personal or community-wide experiences of flooding, fears of future flooding, and associated feelings of guilt to be linked to long-term reductions in SWB (Hudson et al., 2019). O’Brien et al. (2014) also note how long-term stresses such as droughts, not just extreme shocks, can substantially impact SWB, while others show repeated environmental challenges to cumulatively impact SWB by generating a sense of hopelessness (Leviston et al., 2018; Stokols et al., 2009; Walker-Springett et al., 2017). These theories contrast studies which suggest frequent hazards lead to acclimatisation, an acceptance of the risks, and reduced impacts on SWB (Brody et al., 2008; Navarro, 2017).

Land degradation, or forced displacement due to flooding for example, can also lower happiness through “*solastalgia*”, “distress that is produced by environmental change impacting on people while they are directly connected to their home environment” (Albrecht et al., 2007; p.S95). This concept draws on ideas that rural communities’ social identities, relationships (Brown et al., 2021) and spiritual beliefs (Bimrah et al., 2022) are often intertwined with their landscapes and associated ecosystem services (Adams et al., 2020; Schleicher et al., 2018). Therefore, greater place attachment may increase communities’ sensitivity to climate hazards and amplify SWB reductions.

Within rural LMICs, individuals’ livelihoods are often closely intertwined with environmental conditions (Addo et al., 2018b; Shackleton et al., 2014); therefore, unfavourable climates such as prolonged droughts, could restrict the availability of employment for primary sector workers (i.e., crop farmers). Employment is closely related to SWB, as having a “good job” contributes towards personal growth, social network access, status and happiness in Ghana. Being employed and accessing livelihood assets can also reduce individuals’ perceptions of themselves as poor, regardless of objective wealth (Alem et al., 2014; Dzokoto et al., 2019). Limited employment availability may also induce unhappiness by preventing workers from receiving the pride and status attached to fulfilling their intergenerational identities (Brown et al., 2021), practising their indigenous knowledges (Schaafsma, 2019), and undertaking their socially-obligated role of “providers” within the wider family/community (Markussen et al., 2018).

Social capital, defined as “the moral imperative of within-group sharing and redistribution” (Di Falco & Bulte, 2009; p.1), is incorporated within the African worldview of “Ubuntu”, meaning “a person is a person through other persons” (Bangura, 2005; p.31). This worldview promotes respect, an acceptance of differences, a desire to work with others, and the prioritisation of the community over the individual (Nafukho, 2006). Consequently, social capital is a crucial cultural norm within Ghana, which if unfulfilled, could damage community-wide SWB (Dzokoto, 2012).

Interpreting social capital through an environmental lens yields varying results. Firstly, climate hazards may stimulate social cohesion as a coping mechanism (Dussaillant & Guzmán, 2014). Frustrations with surrounding vulnerability or injustice can promote an “in it together” attitude and strengthen community bonds, potentially alleviating climate fears through collective emotional support and the availability of community-funded safety nets (Babcicky & Seebauer, 2017; Calo-Blanco et al., 2017; Jensen & Tiwari, 2021; Jordan, 2015). Therefore, supporting collective adaptive strategies may produce win–win outcomes, with “meaning-focused” activities aimed at overcoming environmental vulnerabilities also generating empowerment and higher SWB (Lawrance et al., 2021). These concepts are central to the “happy farmer” identity commonly portrayed in research and mass media (Kay & Jost, 2003). In contrast, climate pressures could weaken trust and break reciprocal relationships, as declines in productivity increase competition and reduce the resources available to share (Craig et al., 2022; Smith et al., 2024). Furthermore, climate hazards which restrict accessibility and mobility can lower autonomy and isolate individuals from community networks (Beery et al., 2015; Jensen & Tiwari, 2021; Li et al., 2020). For example, flooding which results in roads and paths becoming impassable (Amankwaa & Gough, 2023) can prevent access to meaningful activities such as education; therefore, reducing the capacity to build social resources and lowering SWB by inhibiting self-development (Appiah et al., 2022; Fredrickson & Joiner, 2002; Schueller & Seligman, 2010).

Weakened social ties may further impact SWB by increasing inequality (Adger et al., 2002). Due to the heterogeneity of environmental exposure, sensitivity and adaptive capacity across spatial and temporal scales, less-cohesive communities may experience lower SWB if climate hazards increase the visibility of entrenched inequalities and create more tangible relative comparisons to “better-off” individuals (Kangmennaang et al., 2019). This effect may be exacerbated within well-connected landscapes, where accessibility to road and migration networks with less-vulnerable locations can create higher, potentially unattainable, comparative reference points (Duku et al., 2022; Guillen-Royo & Velazco, 2012).

SWB is influenced by multiple internal and external forces, which can create spatially variable outcomes. These potential SWB determinants are explored within a deltaic landscape exposed to numerous environmental challenges.

## Material & Methods

### Study Area

**Fig. 1:**
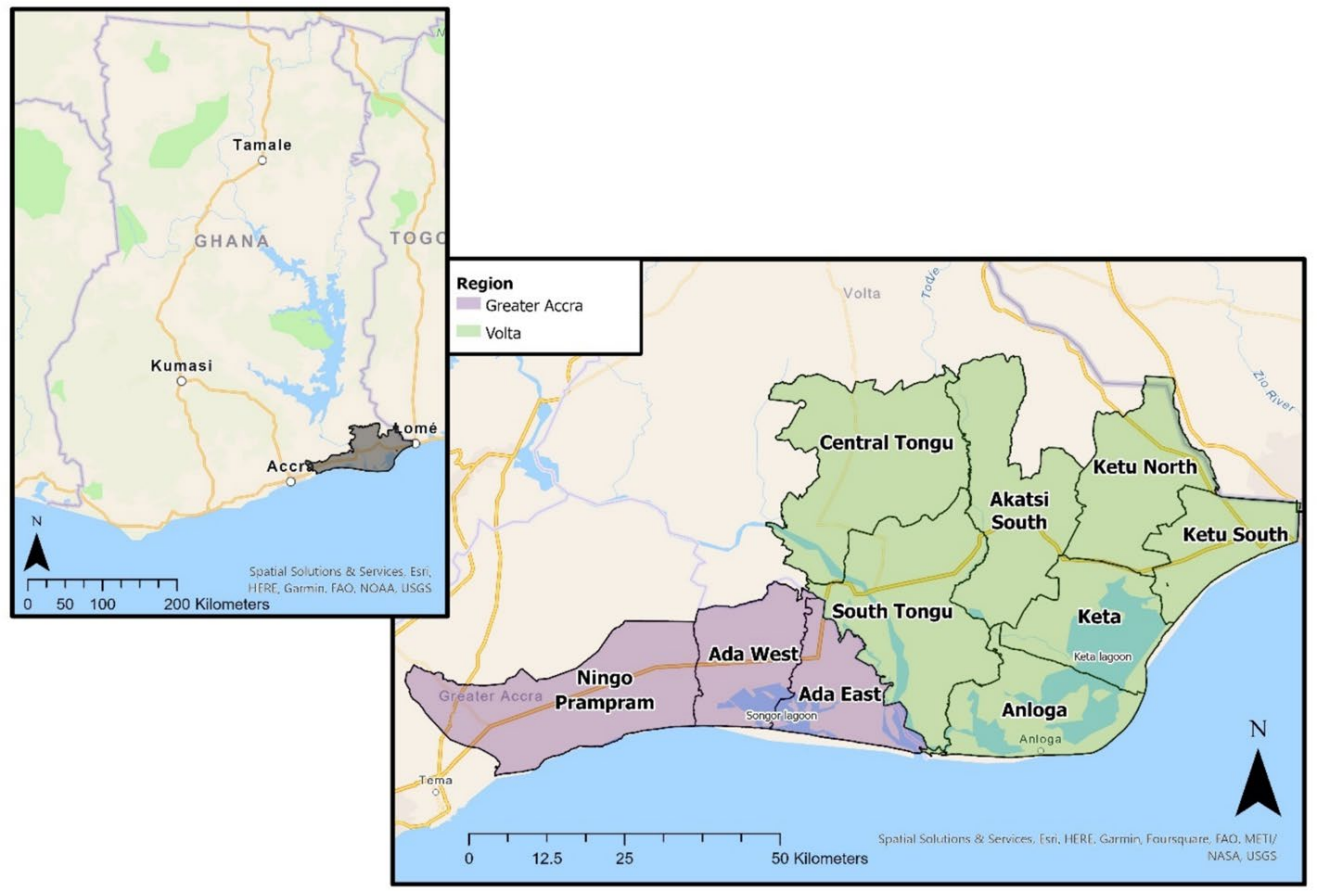
Map of Volta Delta, with regional (Volta and Greater Accra) and district boundaries

Volta Delta is located across two regions, Volta and Greater Accra (Fig. 1), containing 4% of the national population (945,827). Primary sector livelihoods, involving natural resource extraction, contribute most to delta GDP (29%); agriculture (22%) and fishing (7%). Trade, transport, and industry (including salt mining and food processing) contribute 20% of GDP each, and construction contributes 11%. Approximately 1/3 of individuals work in agriculture, which is higher than the proportion of GDP generated (Cazcarro et al., 2018). This disparity is driven by the prevalence of subsistence farming and by low productivity and limited access to technology, which curtail monetary returns (Arto et al., 2020).

The prominence of subsistence agriculture within Volta Delta accentuates the sensitivity of livelihoods and wellbeing to environmental challenges. Key documented climate hazards in global deltas, and Volta Delta, include flooding, coastal erosion, soil and water salinisation, storm surges, cyclones, uncertain monsoon timings and intensified rainfall (Codjoe et al., 2020; Ericson et al., 2006). In particular, sea-level rise along the Accra coast is estimated at 3.3 mm/yr. (1974–2005) (Sagoe-Addy & Addo, 2013), exacerbated by land subsidence (1–2 mm/yr.) (Syvitski, 2008) following extensive groundwater extraction and reduced sediment supply following upstream damming (Addo et al., 2018a). Frequent flooding also contributes towards coastal erosion, estimated at 0.53 m/yr. (1986–2013) (Addo, 2015).

This novel study explores SWB from a landscape perspective in Volta Delta, where existing research has primarily focused on vulnerability and responses to coastal hazards and environmental change. Empirical work has highlighted increasing threats from rainfall variability (Sarku et al., 2020), coastal flooding and erosion (Brempong et al., 2023). Key response strategies identified include flexible farming seasons, irrigation (Sarku et al., 2020), community relocation (Atiglo & Codjoe, 2017; Mattah et al., 2023) and the leveraging of social capital (Mattah et al., 2024). This study investigates how these climatic vulnerabilities and associated adaptive strategies in a fragile environment may impact self-reported SWB outcomes. Previous work in Volta Delta has also explored local understandings of a “good life” (Cannings et al., 2025) and the relationship between OWB and SWB across different locations (Cannings et al., 2024a, b). The present study builds on this existing work by specifically exploring the mechanisms that potentially influence happiness across multiple life domains.

### Data

#### Survey Dataset

Data was obtained from the Deltas, Vulnerability and Climate Change: Migration & Adaptation (DECCMA, 2020) survey. The survey was undertaken face-to-face with household heads (April-June 2016). A two-stage clustered sampling strategy attempted to survey 1,500 households. Households were stratified into five strata based on environmental risk. Fifty enumeration areas,^5^ classified during the 2010 Census, were randomly selected proportional-to-size from the strata. Thirty occupied residential dwellings from each enumeration area were randomly selected. A 91% response rate was achieved (1,364 households). Further information on the survey strategy is available in Atiglo et al. (2022), where the secondary dataset was used to investigate coastal vulnerability.

The survey collected various data, including households’ finances, subjective evaluations, self-reported climate hazards, sociodemographic characteristics, and happiness outcomes. The self-reported hazard variables include shocks (floods & storms) and stresses (drought, salinisation & erosion). Respondents recorded their (i) “exposure”; the hazard is experienced on an annual, or more-frequent, basis, (ii) “environmental impact”; negative impact on housing, health, water and/or food, (iii) “economic impact”; negative impact on economic security and/or crops and livestock.

Happiness data included a global evaluation, “*how happy are you with your life in general?”,* and eight domain variables (Sect. 3.3.1), all measured on five-point Likert scales (very unhappy → very happy). Furthermore, within “subjective evaluations”, two explanatory variables capturing household heads’ “place/community attachment” and “personality cluster” were calculated using ordinal PCA and k-means clustering. Both “subjective evaluation” variables were categorised into 3 groups (low/medium/high). *Place/community attachment* reflects social capital and the strength of relationships between the household head and the local community. The *personality cluster* variable captures the household head’s self-reported positivity, outgoingness and capacity to make changes in their life. See Appendix (A) for details on the calculation of these variables – all appendices are available in the online Supplementary Information.

#### Remote Sensing Data

Multiple remote sensing datasets were collated to create contextual environmental variables.^6^ GPS points for each enumeration area facilitated the spatial join between survey and remote sensing data using *ArcGIS Pro*.

Landcover information was created from LANDSAT-7 30 m resolution images using FAO Land Cover Classifications (Jayson-Quashigah, 2016). The proportion of each landcover classification was calculated for each community buffer.^7^ Existing health studies in Ghana using 2 km buffers (Krefis et al., 2011), the peri-urban/rural mix within Volta Delta (Perez-Heydrich et al., 2013), and the preference for minimal overlap between communities’ associated environments, justified the application of 2 km buffers throughout. Nevertheless, the limitation of individuals working within and accessing environments outside their immediate community is acknowledged (Douxchamps et al., 2015). Further landscape variables were collected from WorldPop (2016) Geospatial data, including distance from major roads, road intersects (Christiaensen et al., 2003) and inland water.^8^ Travel time^9^ from each enumeration area centroid to Accra and district capitals was also constructed using Google API data.

### Methods

#### Outcome Variable: Low Happiness

**Fig. 2:**
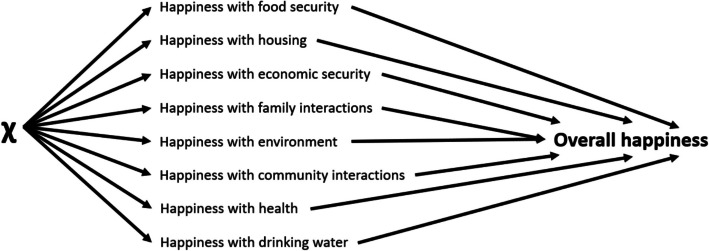
Visualisation of the assumption that overall happiness is an aggregation of happiness with different life domains; adapted from Van Praag et al. (2003)

To address the first research question, “*what are the recorded levels of happiness in Volta Delta?*”, happiness was calculated from an “evaluation” perspective (Table 1) using a “life domains” approach (Rojas, 2006; Vladisavljević and Mentus, 2019). This method assumes happiness with different components additively represents “overall” happiness (Fig. 2). A “life domains” approach was selected over a “global” evaluation due to its decomposable structure enabling respondents to draw on various sources of information and specific experiences, including non-tangible components of wellbeing, which could otherwise be overlooked within abstract "global" responses (Cummins et al., 2003; Kozma et al., 2000; Lent, 2004; Schwarz & Strack, 1999). See Appendix (B) for a comparison between “global” and “life domains” outcomes, and further rationale for selecting the"life domains"approach.

Firstly, an index was calculated using ordinal principal components analysis (PCA)^10^ to agglomerate eight correlating domain scores (Appendix C). The happiness domains, presented in Fig. (2), were based on the question, “*How happy are you with the following dimensions of your life…*?”. These questions were only asked to household heads; therefore, analysis was unable to explore intrahousehold differences.

PCA treats each domain additively, meaning each component consistently contributes towards the index, and is assumed constant across respondents (Van Praag et al., 2003). This approach is common in social research when no individual-level data regarding each domain's self-reported importance is available. See Appendix (C) for further discussion and additional PCA results.

To maintain methodological simplicity and capture an adequate proportion of total variation in the outcome, the first principal component was used as the happiness index; a common method when producing socioeconomic indices (Howe et al., 2008; Martel et al., 2021). Happiness in each domain positively correlated with the overall index (Appendix C). Next, to improve interpretability, the index was grouped into three optimum groups (Vyas & Kumaranayake, 2006) using k-means clustering (De Kruijk & Rutten, 2007) (Appendix D). The standardised happiness index scores ranged from 0.00 to 5.32. The three clusters consisted of low (0.00–2.60), medium (2.61–3.94) and high happiness (3.95–5.32). See Appendix (D) for the distribution of scores across the three clusters.

The clusters were then converted into a binary variable (0 = medium/high happiness, 1 = low happiness) to improve model parsimony, reduce the impact of small index differences which may have resulted from measurement error (Lutz, 2022), and ensure a focus on households with low wellbeing (White, 2010).

“Happiness” and “life satisfaction” are commonly used interchangeably when defining SWB; however, Kahneman and Deaton (2010) note a distinction between everyday happiness, driven by emotions, and long-term satisfaction, incorporating thoughts around one’s life. Nevertheless, Cooke et al. (2016) claim the terms are indistinguishable, and using a single term minimises confusion. Therefore, this study uses “happiness” throughout. Furthermore, despite studies suggesting individuals can comprehend “happiness” and “unhappiness” differently (Kobayashi & Hommerich, 2017; Uchida & Kitayama, 2009), this paper interprets them as two ends of a single continuum; aligning with the survey dataset which presents “unhappy” and “happy” on the same Likert scale.

#### Spatial Distribution

Using *ArcGIS Pro,* a map illustrating the spatial distribution of “low happiness” was produced to provide contextual information and support discussions of modelled results. The proportion of “low happiness” households in each enumeration area was calculated and then categorised into four groups using natural breaks analysis.

#### Regression Modelling

To address the second research question, “*how do climate hazards and landscape characteristics associate with recorded happiness outcomes?”,* a binary logistic regression model was constructed. Multiple independent variables were developed from the available information within secondary remote sensing and survey datasets. This paper focuses on environmental variables, including self-reported climatic hazards and landscape characteristics, yet also recognises the influence of control variables (Table 2). See Appendix (E) for further information, including variable coding.

**Table 2:** Explanatory variable categories and number of variables sourced from DECCMA, WorldPop and various remote sensing sources (see Appendix E for details)

Variable group	Category	No. variables	Data source
Environmental	Climatic hazards (shocks, stresses, shifts/seasonality)	18	DECCMA
	Landscape (remoteness, topography, landcover)	26	Various remote sensing datasets
Control	Household characteristics	6	DECCMA
	Household head characteristics	6	DECCMA
	Adaptation	5	DECCMA
	Assets	4	DECCMA
	Subjective evaluations	2	DECCMA
	Community characteristics	1	WorldPop

Due to the array of multidirectional environment-wellbeing interrelations, such as place attachment (control) potentially being incorporated within environmental happiness (outcome), it is recognised that simultaneity bias may be a source of endogeneity when modelling complex human behaviour.

Following the testing of a multilevel model, a single-level model was constructed due to between-cluster variation being accounted for by inputted variables (Groves et al., 2011). Variables were inputted using a forward stepwise approach in an order based on variable significance and model AIC within preliminary bivariate models: significant environmental, significant control, non-significant environmental, and non-significant control. Interaction terms focusing on environment-control relationships were also tested to capture information on how control characteristics may influence environmental sensitivity and adaptive capacity, and subsequently SWB (Muttarak & Lutz, 2014; Thomas et al., 2019). Households with missing values for variables incorporated in the final model were excluded from the analysis. See Appendix (F) for details on missing data.

Results are presented as odds ratios (exp[βk]), whilst controlling for all other variables in the model. Interaction effects are graphically presented as predicted probabilities, holding all other variables at their mean. Reported results are significant at the 5% level. Model assumptions were fulfilled, including linearity between numerical variables and the log odds of the dependent variable, and no multicollinearity.^11^

As the happiness variable consists of decomposable elements, relationships between significant explanatory variables and specific domains were analysed. This approach can support better-targeted wellbeing initiatives, especially in contexts where communities experience different environmental challenges (Agarwala et al., 2014; Costa, 2003). Chi-square association tests were conducted between each significant model variable and happiness domain (Appendix G). Chi-square analysis does not provide robust evidence of causal relationships; however, association tests were used to help support and structure conceptual discussions.

## Results

### Descriptive Statistics and Overall Rate

Descriptive statistics of the explanatory variables included in the final regression model are presented to provide a profile of the DECCMA sample (Appendix F). Focusing on the environmental variables, cropland coverage within community buffers ranged from 0.0% to 98.4%, whereas the majority of households were located within an enumeration area near a major road (73.6%) and/or containing wetland landcover (70.2%). Furthermore, salinity and storms environmentally impacted 27.6% and 30.4% of households respectively.

**Fig. 3:**
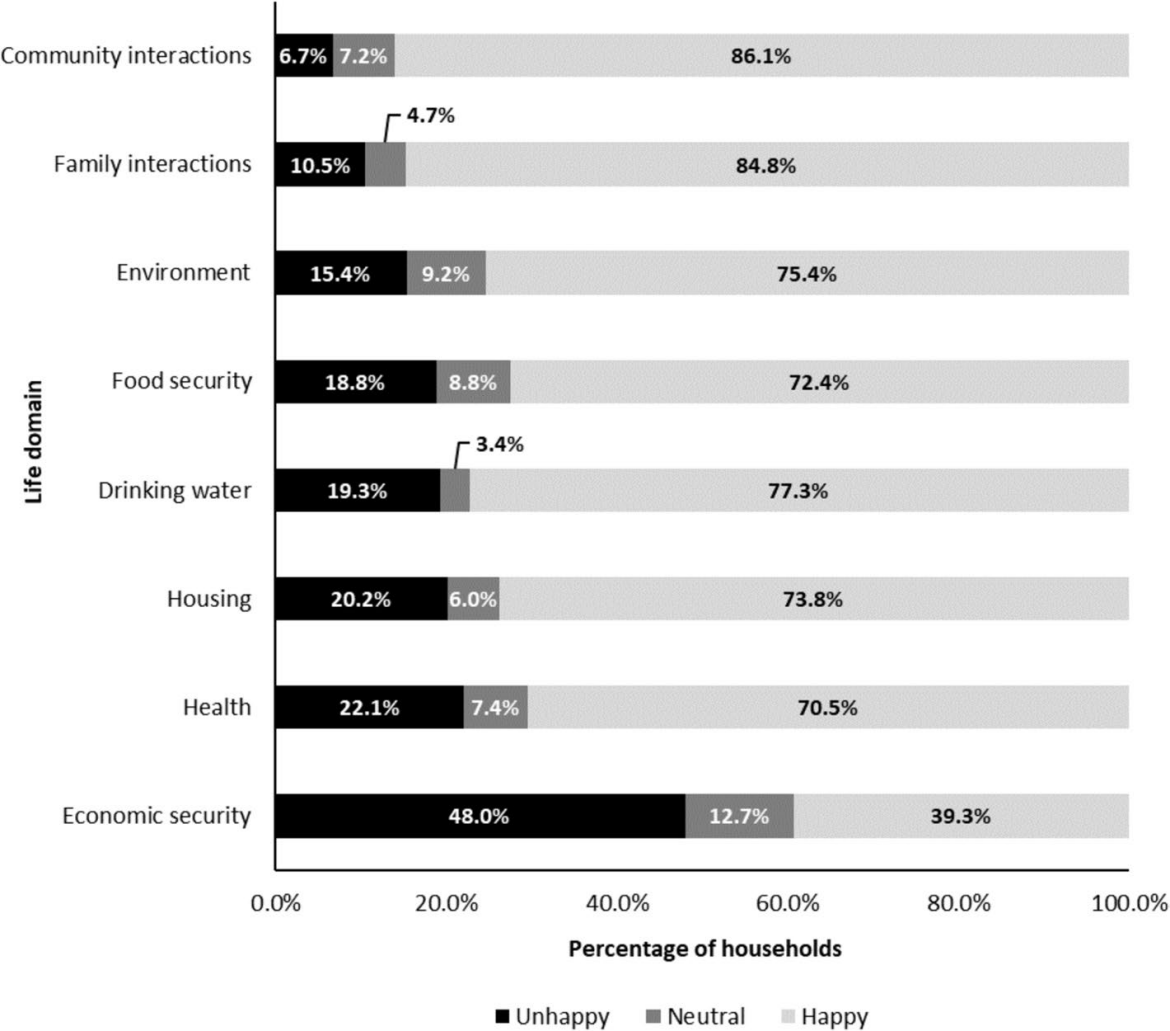
Proportion of households recording unhappiness, neutral responses and happiness with the eight life domains. Moderate and very (un)happy responses are grouped to form three groups from the 5-point Likert scale

Happiness with the different domains varied, with 48.0% (n = 634) moderately/very unhappy with economic security, compared to 6.7% (n = 91) moderately/very unhappy with community interactions (Fig. 3). The binary outcome variable, constructed from the eight domains, categorised 202 households (15%) in the “low happiness” group. See Appendix (H) for a breakdown of the different happiness domains by the overall binary outcome.

### Spatial Distribution

**Fig. 4:**
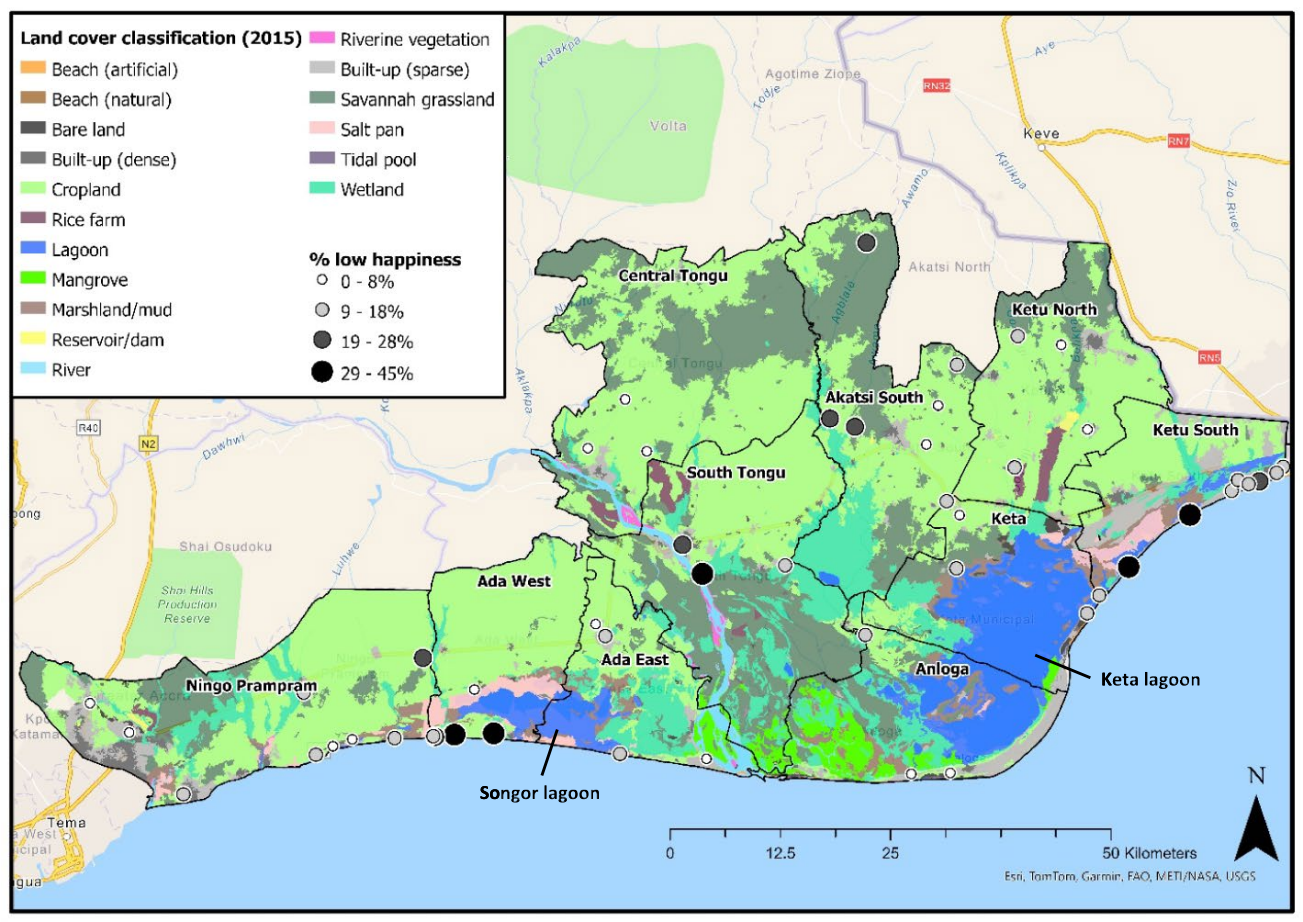
Proportion of households in each enumeration area experiencing low happiness. Landcover information obtained from Jayson-Quashigah (2016) using FAO Land Cover Classifications

High proportions of “low happiness” were concentrated in three areas (Fig. 4): (i) southwest of Songor Lagoon, (ii) coastal areas, close to the Togo border in Ketu South, with salt pans, marshland, and smaller inland water bodies, (iii) South Tongu, including the district capital Sogakope. In contrast, lower proportions are found in Central Tongu, coastal communities near Anloga, west Ningo Prampram, and agricultural areas north of Keta lagoon in Ketu North and Akatsi South.

### Exploratory Analysis

Chi-square tests between each model variable and happiness domain unveiled multiple significant associations (Appendix G). Households impacted by storm surges or salinisation were overrepresented among those reporting low happiness with drinking water, family interactions, community interactions and environment. Storm impacted households also associated with low happiness in food security, housing and health. When exploring landscape characteristics, households in communities with no cropland associated with low happiness in food security, drinking water and family interactions, while those with high cropland coverage were underrepresented among those reporting low happiness with community interactions. In contrast, households in wetland landscapes were overrepresented among those with low happiness in family and community interactions. Household heads in the low personality group were overrepresented among those with low happiness in all domains, with the exception of drinking water, while households with low place attachment associated with low happiness in food security, housing, health, family interactions, community interactions and environment. See Appendix (G) for all chi-square test results.

### Model Results

**Fig. 5:**
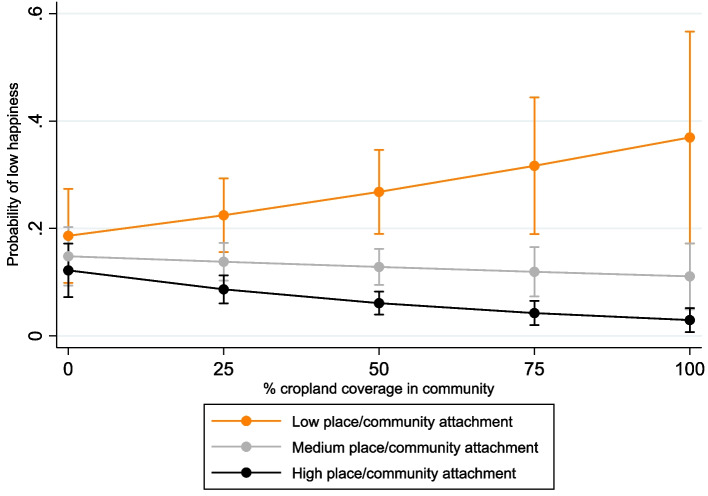
Probability of low happiness by community cropland coverage and place/community attachment (interaction effect)

Table 3 presents the results from the binary logistic regression model. Two significant associations with landcover were recorded; firstly, the presence of wetland increased the odds of low happiness by 88%, whereas every 1% increase in cropland reduced the probability by 0.01 for households with high place/community attachment. Figure (5) illustrates the probability of low happiness to increase as cropland coverage increases for households with low place/community attachment, while the probability lowers for households with medium/high attachment. The probability of low happiness is significantly lower for households with high attachment compared to low attachment at each 25% increment in community cropland coverage. Yet, as cropland coverage increases towards 100% the difference in the probability of low happiness between medium and high place attachment households becomes non-significant. Remoteness from major roads also associated with low happiness, with odds 99% higher for medium-distance (1.7–3.9 km) households than closer locations.

**Table 3:** Binary logistic regression model results, dependent variable: low happiness^+^

Variable	Odds coefficient [exp(β)] (S.E)
Climatic shocks, stresses & shifts	
** Environmental impact from salinity (ref: no impact)**	
Impacted	1.659 (0.341)**
** Environmental impact from storms (ref: no impact)**	
Impacted	*0.769 (0.309)*
Environmental landscape/remoteness	
** Distance from major road (ref: closest to major road)**	
Group 2 (of 3)	1.991 (0.459)***
Group 3 (of 3, furthest from major road)	1.008 (0.346)
** Cropland in 2 km community buffer (%)**	
Percentage coverage	*1.009 (0.006)*
** Wetland in 2 km community buffer (ref: No)**	
Yes	1.880 (0.439)***
Subjective evaluations	
** Place/community attachment (ref: low attachment)**	
Medium attachment	*0.960 (0.344)*
High attachment	*0.854 (0.312)*
** Personality cluster (ref: low score)**	
Medium score	0.606 (0.181)*
High score	0.219 (0.074)***
Physical assets	
** Household roof material (ref: secure, non-natural materials)**	
Non-secure, natural materials	1.749 (0.332)***
Household characteristics	
** Child/adult dependency ratio group (ref: group 1 [lowest])**	
Group 2 – medium	1.415 (0.283)*
Group 3—highest ratio	2.277 (0.625)***
Household head characteristics	
** Employment status of household head (ref: permanent)**	
Non-Permanent	1.539 (0.323)**
Dependant	1.701 (0.496)*
Adaptation	
** Past migrant out of household (ref: no)**	
Yes	1.450 (0.257)**
Interactions	
** Cropland community coverage x Place/community attachment**	
% coverage x Medium attachment	0.987 (0.007)*
% coverage x High attachment	0.976 (0.008)***
** Environmental storm impact x Household head personality cluster**	
Impacted by storm x Medium personality score	2.071 (0.967)
Impacted by storm x High personality score	3.588 (1.845)**
Additional model information	
** Intercept**	0.082 (0.039)***
** No. observations**	1,231
** Log Likelihood**	−439.494

+ *** p < 0.01, ** p < 0.05,* p < 0.1 (significance level)

**Fig.6:**
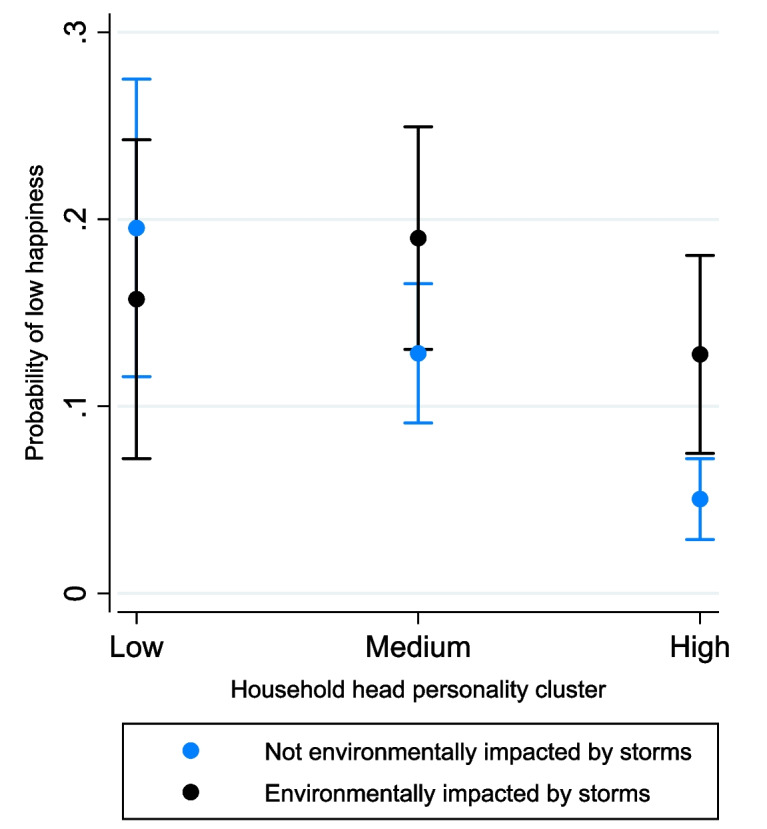
Probability of low happiness by environmental impact from storms and household head personality cluster (interaction effect)

Climate hazards also associated with the SWB outcome, with environmental salinity impacts increasing the odds of low happiness by 66%. Figure (6) also demonstrates that “high personality” households have a higher probability (+ 0.08) of low happiness when environmentally impacted by storms, compared to non-impacted households. The difference is non-significant among those in the “low” and “medium” clusters, as shown by the overlapping confidence intervals. However, amongst non-impacted households, a “high” personality significantly lowered the probability 0.15 and 0.08 compared to “low” and “medium” personality households respectively.

Control variables also explained variation in “low happiness”. Higher odds were recorded for non-permanently employed household heads, within households with non-secure roofing, higher child/adult dependency ratios, and previous migrants.

## Discussion

This section evaluates the overall “low happiness” rate, before exploring regression model results. Model results are interpreted from a landscape perspective to acknowledge the spatial variability of SWB, and how different contextual settings can shape varying outcomes. The key environmental themes from the model include remoteness and connectivity, and agricultural, wetland and coastal landscapes; the latter encompassing impacts from storm surges and salinisation.

### Happiness in Volta Delta

Only 15% of households recorded “low happiness” in the Volta Delta sample, substantially lower than the proportion who self-reported financial stress (87%) or experienced expenditure poverty (37%). The higher level of happiness is hypothesised to have been influenced by survey biases and/or the cultural context. Firstly, Dzokoto (2012) suggests Ghanaians generally have an “optimistic worldview” and attach pride to happiness. Therefore, the result could have been influenced by social desirability bias, where respondents were unwilling to share their struggles to maintain a favourable image in the community (Reisinger, 2022).

Wellbeing is “relational”, meaning it is constructed within a specific time and space (White, 2016). Therefore, it is suggested that the higher happiness levels may have also been influenced by Ghanaian cultural tropes which minimise negative impacts upon SWB and increase self-reported happiness (Dzokoto et al., 2019). For example, Akan cultural teachings encourage individuals to accept life as consisting of “ups and downs” (Dzokoto et al., 2018). Furthermore, there is believed to be a greater acceptance of external constraints, such as political or spiritual powers, which minimises self-blame – contrasting countries such as USA where there is a belief of limitless opportunities and agency. There is also the expectation that Ghanaians are tolerant and accustomed to finding alternative solutions when faced with adversity due to “the social reality of little environmental control and slow socioeconomic and infrastructural development” (Dzokoto, 2012; p.318). Dzokoto et al. (2019) also suggest a laid back sense of time can reduce negative SWB impacts, while lower thresholds for a “good life” in Ghana, such as the contentment with being alive, can boost self-reported happiness; exemplified by the proverb “if the head has not fallen off, you keep putting a hat on it”.

### Agricultural Landscapes

The lower probability of “low happiness” among highly attached, agricultural households (Fig. 5) is supported by Fig. (4), where communities with higher happiness are predominantly situated within cropland landscapes, such as the inland districts of Central Tongu, Akatsi South and Ketu North. These results support the “happy farmer” identity (Markussen et al., 2018), where poorer, rural agricultural communities achieve higher SWB through collective wellbeing conceptualisations (Leviston et al., 2018), greater harmony (Dussaillant & Guzmán, 2014; Schutte et al., 2022), and free access to local resources (Duku et al., 2022). This relationship between happiness and agricultural communities is based upon statistical results, and not a simple perpetuation of the “poor but happy” stereotype which interprets “poor” from a Western capitalist perspective (Kay & Jost, 2003).

Eudaimonic “relatedness” is often pronounced within spaces of ecosystem services such as cropland, as they provide the platform for social interaction (Fagerholm et al., 2016), friendships and knowledge-sharing (Schaafsma & Gross-Camp, 2021). Therefore, agricultural workers are often closely connected to the wider community through their social status and obligated role as providers (Markussen et al., 2018). This hypothesis is supported by exploratory analysis, which showed an association between high cropland coverage and happiness with family and community interactions (Appendix G). Social connections may also strengthen in response to challenging environmental conditions, limited public services and low private investment in remote, rural landscapes (Dussaillant & Guzmán, 2014; Owusu, 2005). Collective actions aimed at combating these broad challenges are capable of boosting SWB by providing a greater sense of agency and empowerment (Brown and Kasser, 2005). Higher levels of bonding social capital^12^ within agricultural landscapes, along with the resulting provision of emotional support and community safety nets, may also increase psychological resiliency and perceived financial security (Addai et al., 2014); thereby contributing towards lower odds of “low happiness”.

The modelled association with place attachment in agricultural areas may also represent how an affiliation with the natural environment can bring happiness in itself, and contribute towards the fulfilment of individuals’ desired livelihoods and identities (Albrecht et al., 2007); particularly amongst subsistence farmers (Bellwood-Howard & Alidu, 2018). Drawing on the “Ubuntu” worldview, Chibvongodze (2016) notes how it not only refers to human–human relationships, but rather a trifecta of human-natural-spiritual connections. Therefore, residing within a productive agricultural landscape with access to natural resources may be encompassed within communities’ broader interpretations of collective wellbeing, and inherently contribute towards happiness.

Despite the association between agricultural landscapes and higher happiness, the model highlights the potential adverse effect of non-permanent employment; with households containing at least one crop farmer being overrepresented among those headed by individuals with non-permanent employment in the DECCMA dataset. The association between non-permanent employment and “low happiness” could be due to individuals’ lesser skills (Leichenko & Silva, 2014) restricting autonomy by limiting alternative livelihoods during environmental stress, entrapping them within fragile and uncertain financial positions (Falco et al., 2018). This potential relationship is supported by the association between non-permanent employment and low happiness with economic security (Appendix G). However, “low happiness” may also exist among skilled workers whose non-permanent employment does not align with their aspirations (Mahmood et al., 2019).

Financial uncertainty within agricultural settings may arise because non-permanent, seasonal employees are often the first to be let go in favour of landowners’ free family labour during low productivity periods (Lipton, 1986; Tambo, 2016). Periodic employment may contribute towards lower SWB by restricting an individual's capacity to master a skill (competence), fulfil their worker identity (Hossain et al., 2016; Ohemeng et al., 2020; Oteng et al., 2021), or perform their social obligations to provide for the family/community (Osei-Tutu et al., 2020). Furthermore, eudaimonic autonomy could be restricted due to underlying climatic uncertainties and non-permanent employees’ dependency upon landowners for work opportunities reducing their financial control (Karabchuk & Soboleva, 2014; Wheatley, 2017). Fewer employment opportunities and reduced financial security may also feedback to lower social capital, and therefore SWB, as monetary resources are a key conduit for forging meaningful, reciprocal relationships within Ghana (Dzokoto et al., 2019). This modelled relationship may also be bidirectional, as lower SWB could limit the capacity for individuals to find permanent employment or increase their productivity (Knapp, 2003).

Irregular employment may also lower happiness by causing health issues, such as depression (Lewchuk et al., 2008); supported by higher proportions of non-permanent households being unhappy with health (Appendix G). An overrepresentation of non-permanent employees unhappy with housing, drinking water and food security also alludes to how seasonal employees may possess insufficient surplus capital or savings to invest in material improvements or adaptive practices, potentially resulting in lower happiness due to the importance of financial wealth and materiality in a Ghanaian “good life” (Coe, 2011; Osei-Tutu et al., 2020).

In contrast, permanent employees' lower odds of “low happiness” may link to their greater financial autonomy, future financial optimism, and ability to plan their expenditure to avoid financial stress during environmental challenges (Heintz, 2004). Furthermore, Alem et al. (2014) suggest employment reduces individuals’ perceptions of themselves as poor, regardless of income, whilst Person et al. (2016) claim the sense of achievement and ability to support the community boosts individuals’ happiness, particularly amongst women.

### Landscape Remoteness & Connectivity

Despite the assumption that more-remote households could experience higher happiness due to their lower awareness of comparatively higher living standards (Ravallion, 2014), the model illustrates no significant difference in the odds between households furthest and closest to major roads. The absence of a significant difference suggests SWB is controlled by alternative mechanisms depending on household connectivity. For example, well-connected households may have greater access to basic needs within district capitals or major towns, such as health and education, with studies suggesting the fulfilment of core needs constitutes the foundation of SWB in LMICs (Bull et al., 2010; Diener et al., 2010; Drakopoulos, 2008; Nanor et al., 2021). In contrast, households remote from public services and markets may conversely benefit from lower relative comparability and greater financial autonomy; for example, “at-the-gate” trading amongst family/friends rather than paying for market transport and intermediaries (Pacillo, 2016).

Nevertheless, model results illustrate households “medium” distances from major roads to have higher odds of “low happiness” than well-connected communities. This result may reflect how the greater visibility of higher urban living standards amongst these households, potentially due to accessible migration networks, increases their aspirations. However, an inability to access the same services and livelihoods as their better-connected counterparts may cause frustrations and lower happiness (Guillen-Royo and Velazco, 2012).

### Coastal Landscapes

A spatial pattern of SWB is illustrated by the higher proportions of “low happiness” amongst coastal communities (Fig. 4), and the higher odds amongst households impacted by salinity and storm surges^13^ (Table 3); with > 80% of households environmentally impacted by salinity located within 1.5 km of the coast. However, this pattern is not unanimous across Volta Delta, with higher happiness recorded in peri-urban coastal communities in Ningo Prampram and Anloga.

Firstly, salinity, which is driven by coastal flooding and/or groundwater inundations (Addo et al., 2018b), is hypothesised to influence SWB in multiple ways; highlighted by an overrepresentation of unhappiness with drinking water, family/community interactions and environment amongst impacted households (Appendix G). Furthermore, due to the less-destructive nature of salinisation compared to flooding, it is under-researched within climate management literature; however, as short-term recovery is harder, it can cause longer-term SWB impacts (Islam et al., 2020).

Salinised aquifers may limit access to potable water for households reliant on groundwater sources, potentially reducing autonomy if water collection from alternative sources reduces the time and energy available for individuals’ desired activities (Guardiola et al., 2013). Furthermore, consuming, cooking or bathing within saline waters (Rahaman et al., 2020) is linked to hypertension (Ahmed et al., 2018; Nahian et al., 2018), strokes (Shammi et al., 2019) and maternal health issues (Desai & Begg, 2008); potentially contributing to lower happiness due to “good health” being a necessity for a “good life” across LMICs (Addai et al., 2014; Dzokoto, 2012; Van Praag et al., 2003).

Storm surges and land degradation due to salinisation may also result in forced migration, and the displacement and fragmentation of families/communities (Addo et al., 2018a; Boateng, 2009; Brown et al., 2021; Mathbor, 2007). These impacts could lower happiness by restricting eudaimonic “relatedness” (Deci & Ryan, 2012) and dampening the SWB benefits associated with functioning in a harmonious, collective community (Ayerakwa et al., 2015). For example, conflicts and social tensions could arise if displaced communities migrate and compete for resources and services with existing communities (Doherty & Clayton, 2011), potentially disrupting reciprocal social ties and threatening individuals’ security (Vladisavljević & Mentus, 2019). These hypotheses tie into the concept of “solastalgia” (Albrecht et al., 2007), as SWB may be reduced if individuals are displaced from areas they are emotionally attached to due to the land's insufficient carrying capacity. This potential mechanism is supported by households impacted by storms and/or salinisation being overrepresented among those unhappy with their environment (Appendix G). Additionally, Sekulova and Bergh (2016) found the experience, or fears of future coastal hazards to create an “emotional unfreedom” (Schaafsma & Gross-Camp, 2021; p.14), where environmental uncertainty generates anxiety and reduces actors’ perceived safety. These negative impacts may contribute to lower happiness by disrupting the “calmness” needed for a “good life” in Ghana (Osei-Tutu et al., 2020).

These ideas surrounding displacement could be further supported by the higher odds of “low happiness” amongst households with past migration experience (Table 3). The relationship between adaptive migration practices and SWB is beyond the scope of this paper; however, the modelled result does emphasise how effective adaptation and migration policy must account for material, as well as “psychological, symbolic and emotional aspects” (Adger, 2010; p.282).

The overrepresentation of environmental unhappiness amongst households impacted by salinity (Appendix G) could reflect how coastal inundation can reduce soil nutrients, as well as crop yields and quality (Lawson et al., 2012; Shrivastava & Kumar, 2015; Williams et al., 2020; Zakari et al., 2014). Jax et al. (2018) suggest “caring for nature” is an innate component of eudaimonic wellbeing; therefore, the damage caused to agricultural or inhabited land could directly reduce SWB. Furthermore, the inability to combat these challenges, and the subsequent feelings of “hopelessness”, could further lower eudaimonic happiness by reducing individuals’ perceived autonomy and ability to control their environment (competence). For example, the autonomy for households to undertake their desired livelihood or adaptive practices could be restrained, such as salinised aquifers curtailing opportunities for irrigation (Hillel & Hatfield, 2005; Leviston et al., 2018). Additionally, if only certain households successfully adapt their livelihoods, existing inequalities could be entrenched, leading to greater relative comparability and community-wide SWB reductions (Kangmennaang et al., 2019).

The modelled relationship between coastal hazards and “low happiness” may also reflect how salinisation and storm surges can damage physical assets such as dwellings (Sagoe-Addy & Addo, 2013). These impacts may induce pessimistic financial outlooks (Ng & Diener, 2014), especially if assets were accumulated as “safety nets” which could be sold to facilitate shock recovery (Zakari et al., 2014). Damaged assets could also lower actors’ self-perceived status or reputation within the community (Dzokoto et al., 2019; Schneider, 2016). For example, Marcus (2006) defines housing as a “mirror of self”, reflecting how one wants to be perceived. Therefore, damaged or low-quality housing may reduce happiness by creating discrepancies between how individuals want to be, and how they are viewed in the community, or by reducing their place attachment (Carroll et al., 2009). These ideas are supported by higher odds of “low happiness” amongst households with low-quality, non-secure roofing materials (Table 3).

Model results also highlight the increased probability of “low happiness” amongst households impacted by storm surges. This result ties into the above discussions regarding salinisation; however, this section will briefly focus on the interaction with household heads’ personality traits (Fig. 6). Amongst non-impacted households a positive personality reduces the probability of “low happiness”, potentially due to “positive” individuals being more willing to take risks and explore new opportunities, which if successful, can reduce environmental vulnerability and financial uncertainties (Cooper et al., 2008; Rashid et al., 2020; Ward et al., 2007). Furthermore, “outgoing” individuals are often more optimistic; therefore, even when encountering challenges their innate traits may enable them to develop subjective resiliencies to objective disadvantages (Rishworth et al., 2020; Seccombe, 2002; Sharpe et al., 2011).

However, despite personality being an underlying driver of happiness (Dowling & Yap, 2012), complexity arises within an environmental context. Figure (6) illustrates the probability of “low happiness” to be significantly higher amongst “high” personality households when environmentally impacted by storms. Initially, this finding is counter-intuitive, as positive personalities are hypothesised to provide psychological barriers to climatic vulnerability (Clare et al., 2017; Jensen & Tiwari, 2021). However, positive individuals may possess higher aspirations, which if unattainable due to climate hazards restricting livelihoods or destroying assets, can dampen happiness (Hudson et al., 2019). This interaction highlights the capacity for climatic hazards to potentially nullify the beneficial influence of personality traits upon SWB.

Furthermore, wellbeing is “relational”, meaning different environments shape individuals’ goals and sense of achievement (White, 2016). Coastal areas in Volta Delta contain more built-up landcover (Fig. 4) and less-vulnerable, non-primary livelihoods. Consequently, despite “resilient” personalities, coastal communities may have “more-to-lose” from flooding and storm damage due to the greater financial security, access to public services and private investment in peri-urban settings (Owusu, 2005). Therefore, reductions to incomes or material assets compared to previous periods of time, or others in the community, may reduce happiness (Ravallion, 2014); especially when acknowledging the often greater emphasis placed on relative positionality when constructing SWB in urban areas (Duku et al., 2022; Guillen-Royo & Velazco, 2012).

### Wetland Landscapes

Wetlands are often associated with objective financial benefits because they provide ideal locations for cultivating important subsistence resources such as rice (Willoughby et al., 2001), and cash crops such as sugarcane. For example, existing research in Volta Delta shows sugarcane farmers to achieve higher incomes and consumption, potentially linked to the emerging use of sugarcane as biofuel (Ahmed et al., 2019). However, previous studies also corroborate model results demonstrating a significant association with lower SWB. Financial opportunities can raise aspirations, which if unattainable due to physical remoteness, limited infrastructure, or climatic uncertainty, may prevent households from achieving their “good life” (Dzokoto et al., 2019). This hypothesis is supported by a study of industrial sugarcane farmers in South Tongu district, with Ahmed et al. (2019) claiming many represented “frustrated achievers” (p.26). Uncertainty regarding market access and fears of climatic hazards, such as floods which previously contributed towards crop failure, prevented these groups from experiencing psychological benefits alongside objective, financial gains.

A specific example of low happiness within wetland landscapes is provided by communities near Songor Lagoon (Fig. 4). Despite residing near diverse livelihood resources such as salt, fish and mangroves, access to such resources is not guaranteed. Originally communities had year-round access to profitable resources, with fishing during the wet season and salt mining in Songor Lagoon during dry periods (Roland et al., 2019). However, the declaration of salt as a state-owned “mineral” in the Minerals and Mining Act (Parliament of the Republic of Ghana, 2006) meant mining could only be undertaken with a licence. The state demand for salt, linked to its use within the emerging petroleum industry (Mensah & Botchway, 2013), resulted in the entire 41,000-acre concession being sold to private ownership (Harvey & Langdon, 2010; Yeboah et al., 2013); continuing Ghana's postcolonial development pathway centred around extracting raw materials (Atta-Quayson, 2023). This shift in ownership prohibited traditional livelihood activities and restricted the capabilities of locals to translate natural capital into financial capital; potentially lowering happiness by preventing individuals from fulfilling their intergenerational identities (Brown et al., 2021). The shift from communal to individual ownership of the lagoon could have also negatively impacted SWB by disrupting collective social norms in rural Ghana (Dzokoto et al., 2019). Additionally, violent conflicts between resistant communities and private police may have also contributed towards lower SWB by threatening individuals’ health and perceived safety (Agbove, 2022; MFWA, 2022).

## Conclusion

This paper contributes towards the understanding of how SWB associates with environmental conditions. Examining these relationships from a landscape perspective in Volta Delta, the model supports the “happy farmer” concept, with a lower probability of “low happiness” amongst agricultural communities with high place/community attachment. This finding contributes towards the growing recognition that happiness can help create a “culture of resilience” (Addai et al., 2014), where higher levels of SWB, theoretically driven by greater reciprocity and collectivism, can mitigate the negative SWB impacts caused by climate hazards within vulnerable rural areas. Furthermore, with the current financial restraints placed upon district governments in Volta Delta, recognising the benefits of improving SWB, and introducing policies to maximise happiness, could be a cost-effective approach to achieving sustainable development. For instance, interventions focused on harnessing community togetherness are less capital and environmentally intensive than income-generating initiatives (Dolan & White, 2007; Michalos, 1997). However, it is imperative to emphasise that happiness should not replace the provision of tangible, material support to vulnerable communities.

In contrast, higher odds of “low happiness” were recorded in peri-urban coastal areas. Lower SWB was attributed to fears of coastal hazards, greater relative comparability, restricted natural resource governance, and potential damage to intergenerational land and livelihoods. The role of relative comparability in lowering SWB may also be reflected in the higher odds of low happiness in areas located at intermediate distances from main roads, where higher living standards in better-connected areas may be visible, yet not accessible. The landscape perspective of SWB also illustrated challenges in wetland locations, potentially attributed to the uncertainty of future environmental conditions or restricted access to key livelihood resources, exemplified by the removal of salt mining rights in Songor lagoon.

It is recognised that SWB could conversely impact environmental conditions; for example, happier individuals may exhibit greater environmental stewardship (Sulemana et al., 2016). However, these relationships were beyond the paper’s scope. Additionally, when reflecting upon our research, it is acknowledged how our position of fortune allowed us to go beyond material wellbeing into the “bonus” psychological needs incorporated within SWB, whereas such concepts may have seemed irrelevant to select communities targeting more immediate, tangible goals (Chumo et al., 2023). However, the focus on SWB within LMICs is justified by evidence showing happiness to influence, and be influenced by, objective wellbeing outcomes.

Individuals'subjective emotions and perceptions govern behaviour, thereby potentially influencing the success of sustainable development policies. Consequently, by illustrating the sensitivity of SWB outcomes to landscape and climatic conditions, this paper advocates for sustainability research to assist policymakers in enhancing the understanding of SWB, and exploring the relationships between happiness and various social, political, and environmental challenges. Integrating wellbeing and sustainability research can support in alleviating the hidden costs associated with low happiness, increasing community buy-in, ensuring local-specific challenges are addressed, and building resiliency within vulnerable socioecological systems. This shift in focus could also broaden the scope of success for Ghanaian development projects, which have traditionally been narrowly focused on top-down economic initiatives.

## Supplementary Information

Below is the link to the electronic supplementary material.

Supplementary file1 (DOCX 170 KB)

## Data Availability

The DECCMA household survey dataset is openly available at; https://data.mendeley.com/datasets/223z53kwnm/1 The landcover classification data is also openly available at; https://www.delta-portal.net/geonetwork/srv/eng/catalog.search;jsessionid=17A16671A3C85ABEA1519F9C670B57E4#/metadata/86b279b8-3cc9-4584-a8bd-9d4300a028df

## Footnotes

1 Rating 0–10 based on their own current life in relation to the worst (0) and best (10) possible life.

2 SDG12 (“ensure sustainable consumption and production patterns”) and SDG13 (“take urgent action to combat climate change and its impacts”).

3 Vulnerability defined by the Development Assistance Research Associates (DARA, 2012) metric which includes “22 indicators across four Impact Areas (Environmental Disasters, Habitat Change, Health Impact, Industry Stress).

4 Mental health indicators are not necessarily interchangeable with happiness outcomes; however, research suggests poor mental health often contributes towards lower SWB (Keller, 2020).

5 Enumeration areas were selected proportional-to-size, and are designed to have similar household counts. Therefore it was assumed households had approximately equal selection probabilities (Groves et al., 2011).

6 Contextual variables are applied to all households within the enumeration area.

7 2 km buffers were drawn around each household in an enumeration area then merged together (using the ArcGIS *dissolve* tool).

8 Inland water distance was obtained from the European Space Agency CCI (Climate Change Initiative) LC (Land Cover project) (Lamarche et al., 2017). Data on proximity to major roads and intersects was taken from OpenStreetMap.

9 Based on a car travelling along recognised road networks as at December 3rd, 2021, at 8:00am.

10 p*rincals* R function.

11 VIF threshold of 4 used to test multicollinearity.

12 Bonding social capital is defined as “strong relationships that develop between people of similar background and interests [who]…provide material and emotional support” (Claridge, 2018; p.2).

13 “*an abnormal rise in seawater level during a storm*” (National Ocean Service, 2022).
