# Supplementary material for: “Happy Farmers” in Volta Delta, Ghana? Exploring the Relationship between Environmental Conditions and Happiness
Source: Soc Indic Res. 2025 Jul 14;179(3):1355–91. doi: 10.1007/s11205-025-03632-8 (PMC12511172; doi:10.1007/s11205-025-03632-8)
Supplement: Supplementary file 1 — Supplementary file1 (DOCX 170 KB) [file 11205_2025_3632_MOESM1_ESM.docx]

**“Happy Farmers” in Volta Delta, Ghana? Exploring the relationship between environmental conditions and happiness**

**Appendix A** Methodological information regarding the calculation of the “place/community attachment” and “household head personality” independent variables

Both “place/community attachment” and “household head personality” variables were constructed using ordinal Principal Components Analysis (PCA) (*princals R-function)* and k-means cluster analysis. The ordinal components within both variables were measured on 5-point Likert scales (Table A1). The first dimension of the PCA analysis was extracted as the index value. Three clusters, using k-means clustering, were consequently constructed to provide low/medium/high values, and to ensure sufficient sample size (>100) within each group.

***Table A1*** *Ordinal variables (measured on 5-point Likert scales) incorporated within the “place/community attachment” and “household head personality” variables*

| **Place/community attachment** | **Household head personality** |
| --- | --- |
| How well do the following statements describe you?: Disagree strongly → Agree strongly | |
| The village is part of my life | I am cheerful and outgoing |
| I want my family and friends to live here  in the future | I am easy to get along with |
| I feel like an outsider in this place (scale reversed) | I am reliable |
| I live here because it is practical | I am relaxed and handle stress well |
| I miss the place when I am not here | I am open to new experiences |
| My friends and family are a good  support for me | I like looking for better ways of  doing things |
| I enjoy being involved in village activities | I feel comfortable making big  decisions (such as changing livelihood or  migrating) |

**Appendix B** Comparison between “life domains” and “global” happiness outcomes

Using the “global” evaluation question, 23% of households were defined as unhappy (moderate/very) in general, whereas 15% of households were categorised as having low “life domains” happiness.

This result disputes existing studies showing greater positivity regarding “global” evaluations than narrower, domain-specific responses (Diener et al., 2000). Despite a significant association between the “global” and “life domains” measures (chi-square association test), contrasting outcomes such as 60% generally unhappy households having higher “life domains” happiness, challenge assumptions that global evaluations mirror aggregated “life domain” responses. These contrasting results support research showing “life domains” happiness to draw on different information and specific experiences, compared to “abstract” global evaluations (Schwarz & Strack, 1999; Kozma et al., 2000; Cummins et al., 2003; Lent, 2004). Therefore, the “life domains” approach is arguably more capable of capturing non-tangible wellbeing components than “global” evaluations. This hypothesis is supported by 78% of generally unhappy households being happy (moderate/very) with “less-tangible” community interactions, yet only 11% being happy with “more-tangible” economic security (Table A2). This distinction is further supported by the PCA loading plot, where the second principal component highlights a differentiation between “tangible” domains of happiness and “less-tangible” domains such as family and community interactions (Figure A3a). Consequently, due to its decomposable nature and greater capacity to incorporate non-material aspects of wellbeing, a “life domains”, rather than a “global”, approach to measuring happiness was selected for further exploratory analysis.

*Table A2. Crosstabulation and chi-square association test between general (global) happiness and happiness with community interactions (Table A2a) and economic security (Table A2b). The 5-point Likert scale was aggregated into a 3-point scale, with moderately/very unhappy and happy combined.*

| *Table A2a* | **Happiness with community interactions** | | |  |
| --- | --- | --- | --- | --- |
| **Happiness in general** | Happy | Neutral | Unhappy | **Total** |
| Happy | 830 (90%) | 50 (5%) | 43 (5%) | **923** |
| Neutral | 94 (78%) | 21 (17%) | 6 (5%) | **121** |
| Unhappy | 251 (78%) | 27 (8%) | 42 (13%) | **320** |
| **Total** | **1,175 (86%)** | **98 (7%)** | **91 (7%)** | **1,364** |
| **Chi-square statistic** | 52.989*** | | | |
|  |  |  |  |  |
| *Table A2b* | **Happiness with economic security** | | |  |
| **Happiness in general** | Happy | Neutral | Unhappy | **Total** |
| Happy | 471 (51%) | 132 (14%) | 320 (35%) | **923** |
| Neutral | 30 (25%) | 23 (19%) | 68 (56%) | **121** |
| Unhappy | 36 (11%) | 18 (6%) | 266 (83%) | **320** |
| **Total** | **537 (39%)** | **173 (13%)** | **654 (48%)** | **1,364** |
| **Chi-square statistic** | 239.208*** | | | |

**Appendix C** Additional PCA information

- Kendall tau-B correlation coefficients for the different life domains incorporated within the happiness index. A value >0.21 is interpreted as a medium strength association, and a value >0.35 as a strong association (Table A3a). The use of PCA is justified by the medium/strong associations between the majority of domains.

| *Table A3a*  **Life domain** | Drinking water | Food security | Housing | Economic security | Health | Community interactions | Family interactions | Environment |
| --- | --- | --- | --- | --- | --- | --- | --- | --- |
| Drinking water |  |  |  |  |  |  |  |  |
| Food security | 0.27 |  |  |  |  |  |  |  |
| Housing | 0.20 | 0.31 |  |  |  |  |  |  |
| Economic security | 0.17 | 0.34 | 0.32 |  |  |  |  |  |
| Health | 0.18 | 0.36 | 0.24 | 0.27 |  |  |  |  |
| Community interactions | 0.12 | 0.24 | 0.27 | 0.15 | 0.17 |  |  |  |
| Family interactions | 0.13 | 0.28 | 0.30 | 0.21 | 0.24 | 0.58 |  |  |
| Environment | 0.18 | 0.25 | 0.30 | 0.28 | 0.22 | 0.38 | 0.28 |  |

- First component loading weights for each life domain. Loadings represent the strength of the correlation with the first component (happiness index). Happiness with each domain positively correlated with the overall index (Table A3b). The first component captured 37% variance.

| *Table A3b*  **Life Domain** | **First Principal Component loading** |
| --- | --- |
| Happiness with food security | +0.658 |
| Happiness with housing | +0.654 |
| Happiness with economic security | +0.640 |
| Happiness with family interactions | +0.637 |
| Happiness with environment | +0.631 |
| Happiness with community interactions | +0.621 |
| Happiness with health | +0.576 |
| Happiness with drinking water | +0.423 |

- PCA loading plot showing the distinction between “tangible” and “less-tangible” domains of happiness. The second principal component primarily captures information on “less-tangible” happiness with family and community interactions. The positive/negative direction of the first component loadings (x-axis) does not affect the interpretation of the plot (Figure A3a).


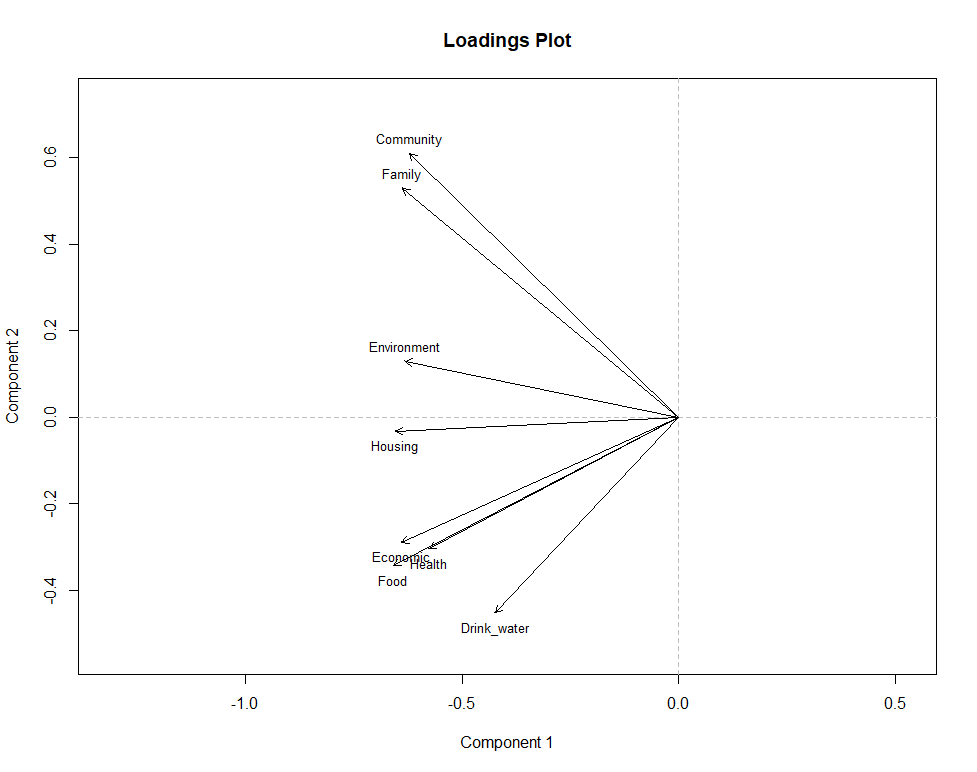


*Figure A3a*

- Discussion on the limitations of using an additive PCA “life domains” approach to measuring happiness

PCA treats each domain additively, meaning each component consistently contributes towards the happiness index. However, this assumption is disputed; for example, happiness in one domain may influence, or be used as a substitute for, another domain (i.e., social relationships dampening financial unhappiness) (Rojas, 2006; Ayerakwa et al., 2015). Furthermore, the contribution of each domain is assumed constant across respondents, termed “ordinal interpersonal comparability” (Van Praag et al., 2003); however, the contributions may differ depending on baseline happiness (Dowling & Yap, 2012; Hodkinson & Martine, 2013). For instance, environmental happiness may contribute more towards overall happiness in historically drought-impacted areas than in favourable environments. In contrast, Van Praag et al. (2003) suggest communities with shared languages possess similar understandings of happiness, minimising differences in interpretation. Nevertheless, without individual-level weighting data for each domain, an additive PCA approach was selected.


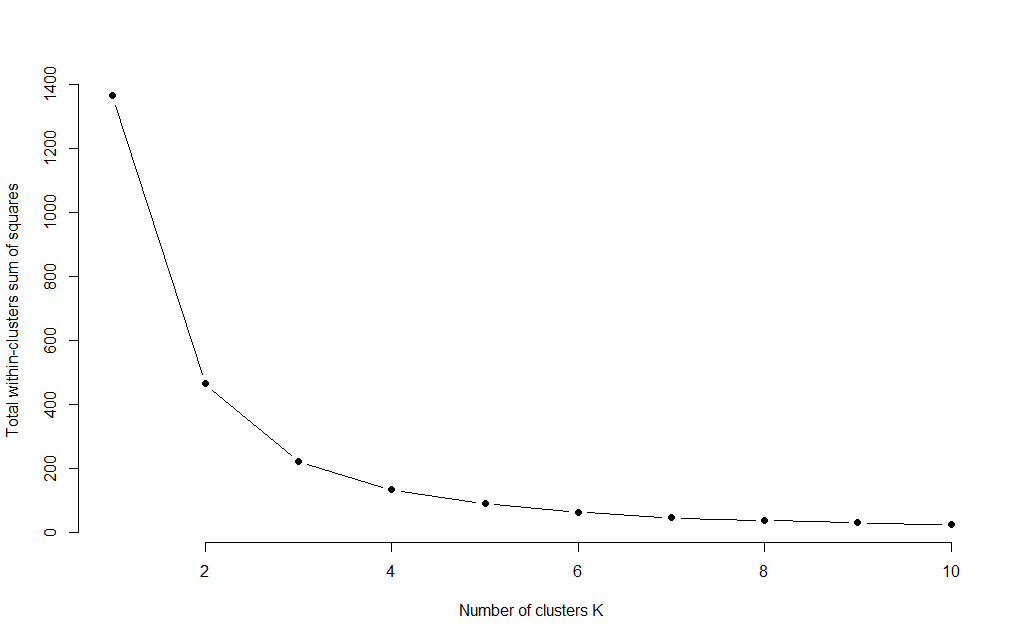
**Appendix D** Visualisation of the “elbow method”, plotting the number of clusters against the total within-clusters sum of squares. The “bend” reflects the optimum number of clusters for analysis. Three “life domains” happiness clusters were chosen.


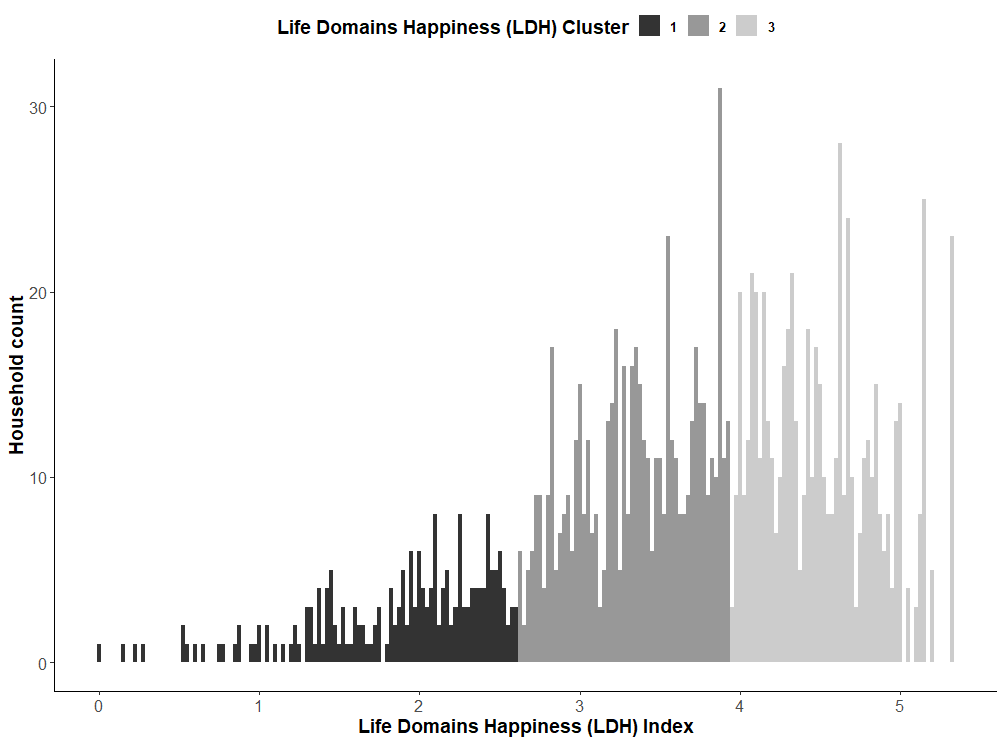
Histogram of the life domains happiness PCA index scores. Categories were defined using k-means clustering. Minimum = 0, Maximum = 5.32, Median = 3.78, Mean = 3.65. Index categorisation; 1 - low (0 - 2.60), 2 - medium (2.61 - 3.94), 3 - high happiness (3.95 - 5.32)

**Appendix E** Variables tested within the regression model, categorised by “environmental” and “control” variables. Information on data sources and how categorisations were defined is also presented.

| **Variable** | **Coding** | **Data source** |
| --- | --- | --- |
| **Environmental variables** |  |  |
| **Climate** | |  |
| **Climate shocks** |  |  |
| Exposure to storms/flooding (annual or more-frequent) | 0: Not exposed to storms/flooding  1: Exposed to storms/flooding | DECCMA |
| Environmental impact from  storms/flooding (negative impact on  housing, health, water and/or food) | 0: Not environmentally impacted by storms/flooding  1: Environmentally impacted by storms/flooding | DECCMA |
| Economic impact from storms/flooding(negative impact on economic security and/or crops and livestock) | 0: Not economically impacted by storms/flooding  1: Economically impacted by storms/flooding | DECCMA |
| **Climate stresses** |  |  |
| Exposure to drought/salinity/erosion  (annual or more-frequent) | 0: Not exposed to drought/salinity/ erosion  1: Exposed to drought/salinity/ erosion | DECCMA |
| Environmental impact from  drought/salinity/erosion (negative  impact on housing, health, water  and/or food) | 0: Not environmentally impacted by drought/salinity/erosion  1: Environmentally impacted by drought/salinity/erosion | DECCMA |
| Economic impact from drought/  salinity/erosion (negative impact on  economic security and/or crops and  livestock) | 0: Not economically impacted by drought/salinity/erosion  1: Economically impacted by drought/salinity/erosion | DECCMA |
| **Climate shifts/seasonality** |  |  |
| Change in rainfall over the last 5 years | 0: Stayed the same 1: Changed (increased/decreased/changed another way) | DECCMA |
| Change in rainfall timing over the last 5 years | 0: Stayed the same 1: Earlier rainy season 2: Later rainy season | DECCMA |
| Change in temperature over the last 5 years | 0: Stayed the same 1: Changed (increased/decreased/changed another way) | DECCMA |
| **Landscape** | |  |
| **Remoteness** |  |  |
| Distance to coast | 0: 0-<10km  1: 10-20km  2: 20km+  *(10km increments)* | WorldPop (US NASA Shuttle Radar Topography Mission (SRTM)) |
| Distance to inland water (km) | Numeric (km) | WorldPop (European Space Agency CCI) |
| Travel time to Accra | 0: 1-2 hours  1: 2-3 hours  2: 3+ hours  *(1 hour increments)* | Produced using Google API data |
| Travel time to district capital | 0: 0-10 mins  1: 11-20mins  2: 21-30 mins  3: 30+ mins (reference)  *(10-min increments)* | Produced using Google API data |
| **Variable** | **Coding** | **Data source** |
| Distance to major road | 0: 0-1.77km  1: 1.78-3.93km  2: 3.93km+  *(natural breaks)* | WorldPop (OpenStreetMap) |
| Distance to road intersect | 0: 1-3.15km  1: 3.16-6.3km  2: 6.3km+  *(natural breaks)* | WorldPop (OpenStreetMap) |
| Region | 0: Volta 1: Greater Accra | x |
| **Topography** |  |  |
| Elevation | 0: 0-10.5masl  1: 10.6-27masl  2: 27masl +  *(natural breaks)* | WorldPop (US NASA Shuttle Radar Topography Mission (SRTM)) |
| Slope | 0: Under 1°  1: 1° or more  *(logical threshold)* | WorldPop (US NASA Shuttle Radar Topography Mission (SRTM)) |
| **Landcover** |  |  |
| EVI vegetation index (2016) | *3-group categorisation (natural breaks)*  0: 0.113-0.198  1: 0.199-0.269  2: 0.270+ | NASA (MODIS) |
| EVI % change (2006-16) | 0: High decrease (-50% to -15%)  1: Low decrease (-14% to 0%)  2: Low increase (0% to 17%)  3: High increase (18% to 52%)  *(two natural breaks above/below 0%)* | NASA (MODIS) |
| EVI % change (2011-16) | 0: High decrease (-55% to -19%) 1: Low decrease (-19% to 0%) 2: Low increase (0% to 25%) 3: High increase (26% to 63%)  *(two natural breaks above/below 0%)* | NASA (MODIS) |
| EVI % change (2015-16) | 0: High decrease: (-57% to -36%) 1: Medium decrease: (-35% to -19%) 2: Low increase: (-18% to 0%) 3: Increase: (Over +0%)  *(natural breaks below 0%)* | NASA (MODIS) |
| Cropland | *Numeric value*  % within 2km community buffer | LANDSAT-7, FAO Classifications (Jayson-Quashigah, 2016) |
| Change in cropland (2001-15) | *3-group categorisation (logical groups)*  0: No change (-1% > < +1%)  1: Decrease (<-1%)  2: Increase (>+1) | LANDSAT-7, FAO Classifications |
| Savannah grassland | *Numeric value*  % within 2km community buffer | LANDSAT-7, FAO Classifications |
| Riverine vegetation | 0: Riverine vegetation not present in community buffer  1: Riverine vegetation present in community buffer | LANDSAT-7, FAO Classifications |
| Mangrove | 0: Mangrove not present in community buffer  1: Mangrove present in community buffer | LANDSAT-7, FAO Classifications |
| Wetland | 0: Wetland not present in community buffer  1: Wetland present in community buffer | LANDSAT-7, FAO Classifications |
| Bare land | 0: Bare land not present in community buffer  1: Bare land present in community buffer | LANDSAT-7, FAO Classifications |
| **Variable** | **Coding** | **Data source** |
| Marshland | 0: Marshland not present in community buffer  1: Marshland present in community buffer | LANDSAT-7, FAO Classifications |
| Lagoon | 0: Lagoon not present in community buffer  1: Lagoon vegetation present in community buffer | LANDSAT-7, FAO Classifications |
| Beach | 0: Beach not present in community buffer  1: Beach present in community buffer | LANDSAT-7, FAO Classifications |
| Salt pan | 0: Salt pan not present in community buffer  1: Salt pan present in community buffer | LANDSAT-7, FAO Classifications |
| Built-up land | *3-group classification (natural breaks)*  0: 0% - 16%  1: 17% - 38%  2: >39% | LANDSAT-7, FAO Classifications |
| % change in built-up land (2001-15) | 0: No change  1: Decrease  2: Increase | LANDSAT-7, FAO Classifications |
| **Control variables** |  |  |
| **Household characteristics** |  |  |
| Household size | *5-group categorisation (logical groups)*  1: 1 person  2: 2-3 people  3: 4-5 people  4: 6-7 people  5: 8+ people | DECCMA |
| Dependants in households | 0: 0  1: 1 to 2  2: 3 to 4  3: 5+  *(logical groups)* | DECCMA |
| Child:adult dependency ratio | *3-group classification (logical groups)*  0: 0 - 0.66  1: 0.67 - 2  2: 2+ | DECCMA |
| Proportion of females | *Numeric value*  % females in household | DECCMA |
| Religion | 0: Christian  1: Non-Christian (Islam, Buddhism, Hinduism, Traditional) | DECCMA |
| Livelihood cluster | 0: Salaried employee/business owner  1: Fishing/trade/transport/construction  2: Crop farmer  *(MCA and cluster analysis)* | DECCMA |
| **Household head characteristics** |  |  |
| Years of schooling | 0: No Schooling  1: Below-basic education  2: Basic education  3: Above-basic education | DECCMA |
| How long have you lived in the village? | 0: Entire life  1: Migrated more than 10 years ago  2: Migrated less than 10 years ago | DECCMA |
| Marital status | 0: Married or cohabitating  1: Never married  2: Previously married | DECCMA |
| Employment status | 0: Permanent  1: Non-permanent  2: Dependent (unemployed, student or retired) | DECCMA |
| Sex | 0: Male  1: Female | DECCMA |
| Age *(quartiles)* | 0: 18-33 years  1: 34-45 years  2: 46-60 years  3: 60+ years | DECCMA |
| **Variable** | **Coding** | **Data source** |
| **Adaptation** |  |  |
| Any form of adaptation (last 5 years) | 0: No adaptation in the last 5 years  1: At least one form of adaptation in the last 5 years | DECCMA |
| Current migrant outside household | 0: No migrant currently outside household  1: Migrant currently outside household | DECCMA |
| Past migrant outside household | 0: No migrant previously outside household  1: Migrant previously outside household | DECCMA |
| Intention to migrate? | 0: No intention  1: Intention to migrate | DECCMA |
| Migration evaluation | 0: Not helpful/neither helpful nor unhelpful  1: Helpful | DECCMA |
| **Assets** |  |  |
| Latrine type | 0: Flushing latrine  1: Pit latrine, Public latrine or KVIP  2: No facility | DECCMA |
| Drinking water source | 0: Piped/tubewell/standpipe  1: Dug well/open source | DECCMA |
| Roof material | 0: Non-secure  1: Secure | DECCMA |
| Homeownership | 0: Not owned (mortgaged, renting, squatting)  1: Owned | DECCMA |
| **Subjective evaluations** |  |  |
| Place/community attachment | 0: Low  1: Medium  2: High | DECCMA |
| Personality | 0: Low  1: Medium  2: High | DECCMA |
| **Community characteristics** |  |  |
| Population density (2016) | 0: Low (74 - 369 people/km^2^)  1: Medium (370 - 877 people/km^2^)  2: High (878 - 2702 people/km^2^)  *(natural breaks)* | WorldPop |

**Appendix F** Descriptive statistics of the explanatory variables incorporated within the binary logistic regression model (see Table 3).

| **Variable group** | **Variable** | **Descriptive statistics** |
| --- | --- | --- |
| Environmental | Cropland in community | Mean – 41.3%  Median – 42.8%  Min – 0.0%  Max – 98.4% |
|  | Environmental impact from salinity | Not impacted - 921 (67.5%)  Impacted – 376 (27.6%)  *Missing – 67 (4.9%)* |
|  | Environmental impact from storms | Not impacted – 886 (65.0%)  Impacted – 415 (30.4%)  *Missing – 63 (4.6%)* |
|  | Wetland landcover present in community | Not present – 407 (29.8%)  Present – 957 (70.2%) |
|  | Distance from major road | Low [0-1.77km] – 1,004 (73.6%)  Medium [1.78-3.93km] – 195 (14.3%)  High [3.93km+] – 165 (12.1%) |
| Control | Roof material | Non-secure [inc. natural materials] – 626 (45.9%)  Secure [inc. cement/tiles] – 738 (54.1%) |
|  | Place/community attachment cluster | Low – 195 (14.3%)  Medium – 522 (38.3%)  High – 647 (47.4%) |
|  | Personality cluster | Low – 182 (13.3%)  Medium – 560 (41.1%)  High – 622 (45.6%) |
|  | Child/adult dependency ratio | Low [0 - 0.66] – 880 (64.5%)  Medium [0.67 – 2] – 359 (26.3%)  High [2+] – 125 (9.2%) |
|  | Past migrant outside household | No past migrant – 723 (53.0%)  Past migrant – 641 (47.0%) |
|  | Household head employment status | Permanent – 937 (68.7%)  Non-permanent – 281 (20.6%)  Dependant – 125 (9.2%)  *Missing – 21 (1.5%)* |

|  | **Unhappiness (moderate/very) with life domain…** | | | | | | | |
| --- | --- | --- | --- | --- | --- | --- | --- | --- |
| **Explanatory variable** | **Food security** | **Housing** | **Economic security** | **Health** | **Drinking water** | **Family interactions** | **Community interactions** | **Environment** |
| Cropland in community  (4 groups) | **↑**  No/low/medium coverage  (groups 1-3/4) |  | **↑**  Medium coverage  (group 3/4) |  | **↑**  No coverage  (group 1/4) | **↑**  No coverage  (group 1/4) | **↓**  High coverage  (group 4/4) |  |
| Environmental impact from salinity |  |  |  |  | **↑**  Impacted | **↑**  Impacted | **↑**  Impacted | **↑**  Impacted |
| Environmental impact from storms | **↑**  Impacted | **↑**  Impacted |  | **↑**  Impacted | **↑**  Impacted | **↑**  Impacted | **↑**  Impacted | **↑**  Impacted |
| Wetland landcover present in community |  | **↑**  Wetland communities |  |  |  | **↑**  Wetland communities | **↑**  Wetland communities | **↑**  Wetland communities |
| Distance from  major road  (3 groups) | **↑**  Medium distance (group 2/3) |  | **↑**  Medium distance (group 2/3) | **↑**  Medium distance (group 2/3) | **↑**  Medium distance (group 2/3) |  |  | **↑**  Highest distance (group 3/3) |
| Roof material | **↑**  Non-secure roofing | **↑**  Non-secure roofing | **↑**  Non-secure roofing |  |  |  |  |  |
| Place/community attachment cluster  (3 clusters) | **↑**  “Low/medium” attachment  (groups 1-2/3) | **↑**  “Low” attachment (group 1/3) |  | **↑**  “Low” attachment (group 1/3) |  | **↑**  “Low” attachment (group 1/3) | **↑**  “Low” attachment (group 1/3) | **↑**  “Low” attachment (group 1/3) |
| Personality cluster  (3 clusters) | **↑**  “Low” personality (group 1/3) | **↑**  “Low” personality (group 1/3) | **↑**  “Low” personality (group 1/3) | **↑**  “Low” personality (group 1/3) |  | **↑**  “Low” personality (group 1/3) | **↑**  “Low” personality (group 1/3) | **↑**  “Low” personality (group 1/3) |
| Child/adult dependency ratio |  |  | **↑**  High ratio  (group 3/3) |  |  |  | **↑**  High ratio  (group 3/3) |  |
| Past migrant outside household | **↑**  Past migrant household |  |  | **↑**  Past migrant household | **↑**  Past migrant household | **↑**  Past migrant household | **↑**  Past migrant household |  |
| Household head employment status | **↑**  Non-permanent/  dependant | **↑**  Non-permanent | **↑**  Non-permanent/  dependant | **↑**  Non-permanent/  dependant | **↑**  Non-permanent |  |  |  |

**Appendix G** Chi-square test results between significant explanatory variables in the regression model (Table 3) and each individual life domain. Associations significant at the 5% level are presented^^[[1]](#footnote-1)^^. Standardised residuals are used to determine whether low happiness is over (↑) or underrepresented (↓) by each explanatory variable. The five-point Likert responses for each domain were aggregated into three groups: unhappy (moderate/very), neutral, and happy (moderate/very).

**Appendix H** Breakdown of happiness with each life domain - unhappy (moderate/very), neutral, and happy (moderate/very) – by overall binary happiness classification (low & medium/high life domains happiness). 202 households defined as “low happiness”, and 1,162 households with “medium/high” happiness.

|  |  | **Number of households (%)** | | |
| --- | --- | --- | --- | --- |
| **Life domain** | **Binary happiness classification** | **Unhappy** | **Neutral** | **Happy** |
| Community interactions | Low | 62 (31%) | 35 (17%) | 105 (52%) |
|  | Medium/high | 29 (3%) | 63 (5%) | 1,070 (92%) |
| Family interactions | Low | 82 (41%) | 26 (13%) | 94 (46%) |
|  | Medium/high | 61 (5%) | 38 (3%) | 1,063 (92%) |
| Environment | Low | 103 (51%) | 36 (18%) | 63 (31%) |
|  | Medium/high | 107 (9%) | 90 (8%) | 965 (83%) |
| Food security | Low | 131 (65%) | 25 (12%) | 46 (23%) |
|  | Medium/high | 126 (11%) | 95 (8%) | 941 (81%) |
| Drinking water | Low | 99 (49%) | 12 (6%) | 91 (45%) |
|  | Medium/high | 164 (14%) | 34 (3%) | 964 (83%) |
| Housing | Low | 135 (67%) | 14 (7%) | 53 (26%) |
|  | Medium/high | 140 (12%) | 68 (6%) | 954 (82%) |
| Health | Low | 127 (63%) | 15 (7%) | 60 (30%) |
|  | Medium/high | 174 (15%) | 86 (7%) | 902 (78%) |
| Economic security | Low | 192 (95%) | 7 (3%) | 3 (2%) |
|  | Medium/high | 462 (40%) | 166 (14%) | 534 (46%) |

1. Note, cropland coverage is a numerical value (%) in the regression model, yet here it is categorised into four clusters, using natural breaks, to facilitate the categorical association test. [↑](#footnote-ref-1)
